# aKNNO: single-cell and spatial transcriptomics clustering with an optimized adaptive k-nearest neighbor graph

**DOI:** 10.1186/s13059-024-03339-y

**Published:** 2024-08-01

**Authors:** Jia Li, Yu Shyr, Qi Liu

**Affiliations:** 1 Department of Biostatistics, Vanderbilt University Medical Center, Nashville, TN 37203 USA; 2 Center for Quantitative Sciences, Vanderbilt University Medical Center, Nashville, TN 37203 USA

**Keywords:** Single-cell and spatial transcriptomics, Clustering, Rare cells, Adaptive k-nearest neighbors

## Abstract

Typical clustering methods for single-cell and spatial transcriptomics struggle to identify rare cell types, while approaches tailored to detect rare cell types gain this ability at the cost of poorer performance for grouping abundant ones. Here, we develop aKNNO to simultaneously identify abundant and rare cell types based on an adaptive *k*-nearest neighbor graph with optimization. Benchmarking on 38 simulated and 20 single-cell and spatial transcriptomics datasets demonstrates that aKNNO identifies both abundant and rare cell types more accurately than general and specialized methods. Using only gene expression aKNNO maps abundant and rare cells more precisely compared to integrative approaches.

**Supplementary Information:**

The online version contains supplementary material available at 10.1186/s13059-024-03339-y.

## Background

Single-cell and spatial transcriptomics provide an unprecedented opportunity to navigate cellular landscape in complex tissues [1–4]. To understand cellular heterogeneity, one essential step is to define cell types through unsupervised clustering based on transcriptome similarity [5]. A number of clustering methods have been developed, most of which are generic algorithms adapted for single-cell transcriptomics analysis, such as *k*-means, hierarchical, density-based [6], and community-detection-based clustering [7]. For example, RaceID [8], SIMLR [9], and SC3 [10] refine *k*-means for robust cell clustering. CIDR [11], BackSPIN [12], and pcaReduce [13] extend hierarchical clustering to improve grouping ability on single-cell transcriptomics. Phenograph [14], Seurat [15], and scanpy [16] apply community-detection methods to define cell clusters. Although those typical methods achieved good performance in identifying abundant cell types, they all face the challenge of detecting rare ones.

To address the challenge, several approaches have been specifically designed or tailored to detect rare cell types, such as RaceID [8], GiniClust [17], GiniClust3 [18], FiRE [19], and GapClust [20]. RaceID supplements k-means clustering with outlier detection to identify rare cell types [8]. GiniClust selects genes with high Gini index and then discovers rare cells based on density-based clustering [17]. GiniClust3 extends the method to identify both abundant and rare cell clusters using a cluster-aware, weighted ensemble approach [18]. FiRE uses the Sketching technique to assign a rareness score to each cell [19]. GapClust captures the abrupt local distances change to find rare cell clusters [20]. Those methods either only target rare cells but ignore abundant ones or gain the ability of identifying rare cells at the cost of poorer performance for clustering abundant ones [21].

Here, we propose aKNNO, which builds an optimized adaptive *k*-nearest neighbor graph for community-based detection. Compared to the traditional *k*-nearest neighbor (*k*NN) graph requiring a prespecified and fixed *k* for all cells, aKNNO chooses *k* adaptively for each cell based on its local distance distribution. The adaptive strategy enables the accurate detection of both abundant and rare cell types in a single run. The optimization step gets the most of the adaptive strategy and further improves rare cells identification. Benchmarked on 38 simulated and 17 single-cell transcriptomics datasets, aKNNO outperformed other methods for rare cells identification without scarifying the performance on abundant cells clustering. Applied on three spatial transcriptomics datasets, aKNNO stereotyped fine-grained anatomical patterns using gene expression alone, some of which were even missed by those methods integrating gene expression, spatial locations, and histology image.

## Results

### Overview of aKNNO

The community-detection methods have become increasingly popular, particularly for analyzing large single-cell transcriptomics datasets [21]. They first construct a *k*NN graph by connecting each cell to its nearest *k* cells measured by transcriptome similarity and then group cells with dense connection. The choice of *k* has great impact on the clustering performance. A large *k* may generate phony connections between rare cells and cells in other clusters, while a small *k* may lead to overclustering of abundant cell types due to dominance of local variances. Cell populations in single-cell data are generally highly imbalanced, including both abundant and rare cells. Therefore, the traditional *k*NN graph using a universal *k* for all cells is unable to capture the inherent cellular structure accurately.

**Fig. 1:**
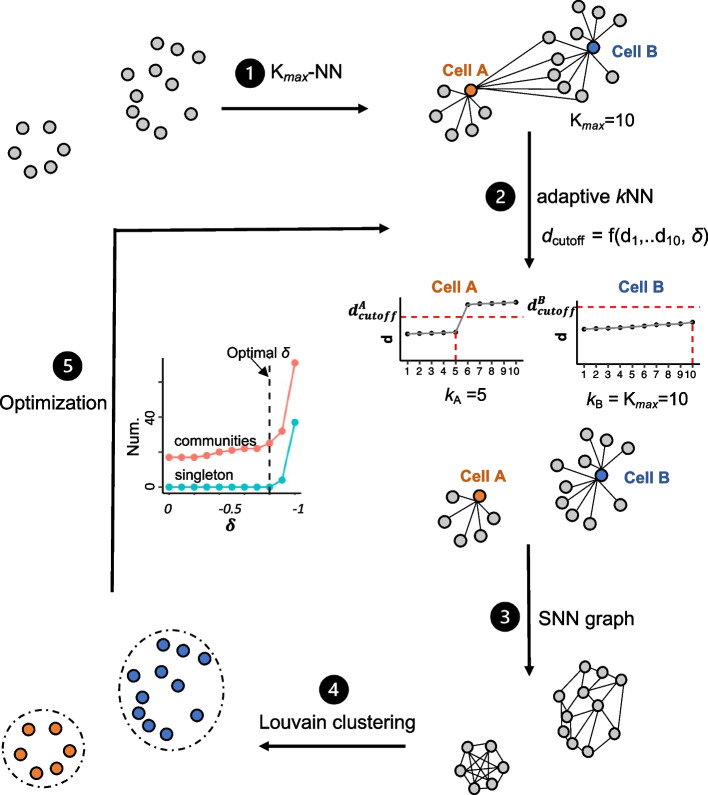
Overview of aKNNO. aKNNO includes five steps: (1) calculating the *K*_*max*_-nearest neighbors for all cells, (2) choosing *k* adaptively for each cell based on its local distance distribution, (3) building the shared nearest neighbor graph, (4) clustering based on Louvain community-detection, (5) optimizing the hyperparameter $\delta$ by grid search

Instead of using a single *k* value for all cells, aKNNO chooses *k* adaptively for each cell based on its local distance. It automatically assigns a small *k* for rare cells to remove spurious long-range connections and connect only true nearest neighbors and a large *k* for abundant cells to balance local and global variances. We used a toy example to illustrate how *k* is chosen adaptively (Fig. 1). In the example, *K*_*max*_ is set to 10, meaning that the choice of *k* ranges from 1 to 10. For each cell, aKNNO finds its 10-nearest neighbors and sorts the distance in an ascending order (*d*_1_ < *d*_2_ < … < *d*_10_). *k* = *K*_*max*_ = 10 if *d*_10_ < *d*_cutoff_; otherwise, *k* is chosen if *d*_*k*_ < *d*_cutoff_ and *d*_*k*+*1*_ $\ge$ *d*_cutoff_. *d*_cutoff_ is determined by 10-nearest distances of the cell and tuned by a hyperparameter $\delta$ (*d*_cutoff_ = *f*(*d*_1_,..*d*_10_,$\delta$)). As an example, cell A comes from a rare cluster containing only six cells; thus, its 10-nearest distances increase dramatically from *d*_5_ to *d*_6_, leading to its *d*_*5*_ < ${d}_{cutoff}^{A}$ and *d*_6_ > ${d}_{cutoff}^{A}$. Therefore, *k* of 5 is chosen for the cell A (*k*_A_ = 5). As another example, cell B belongs to an abundant cell type, where its 10-nearest distances have a slow increase from *d*_1_ to *d*_10_, resulting in its *d*_10_ < ${d}_{cutoff}^{B}$. In this case, *k* of 10 is selected for the cell B (*k*_B_ = 10). In this way, aKNNO assigns the adaptive and optimal neighbors for each cell. To improve the robustness, the adaptive nearest-neighbor graph is reweighted based on the shared nearest neighbors of pairs of cells (SNN). Finally, Louvain community detection method is applied on the shared nearest neighbor graph to identify clusters (Fig. 1). The hyperparameter $\delta$ controls the sensitivity of aKNNO to the local distance change. aKNNO performs a grid search to find the optimal $\delta$ that balances the sensitivity and specificity of rare cluster identification (Fig. 1).

### Performance of aKNNO in simulated datasets

We first compared the performance of aKNNO to the traditional *k*NN-based method in the Seurat [15] (denoted as KNN) using simulated datasets with ground truth cell-type identity. To make a fair comparison, aKNNO and KNN processed the data exactly the same way except that aKNNO clustered cells based on an adaptive *k*-nearest neighbor graph while KNN grouped cells from a *k*NN graph with a fix *k* for all cells (default *k* = 20, details in the “Methods” section). We generated simulated datasets using a public single-cell RNA-seq dataset with 2700 peripheral blood mononuclear cells (PBMC3k) and nine cell types. We simulated two settings, each of which contained two abundant and one rare cell types. In one setting, the two abundant cell types were B cells (*n* = 200) and naïve CD4 T (*n* = 200), while the rare cell type was nature killer (NK) cell. In the other setting, the two abundant cell types were the same, while the rare cell type was CD14 + monocyte. The first setting was more challenging than the second since its rare cells (NK) were similar to one of abundant cell types (naïve CD4 T), indicating that those rare cells were more likely to be hidden by abundant ones. We simulated 19 scenarios for each setting with the number of rare cells ranging from 2 to 20, and we generated 50 datasets for each scenario by random sampling cells from the PBMC3k.

**Fig. 2:**
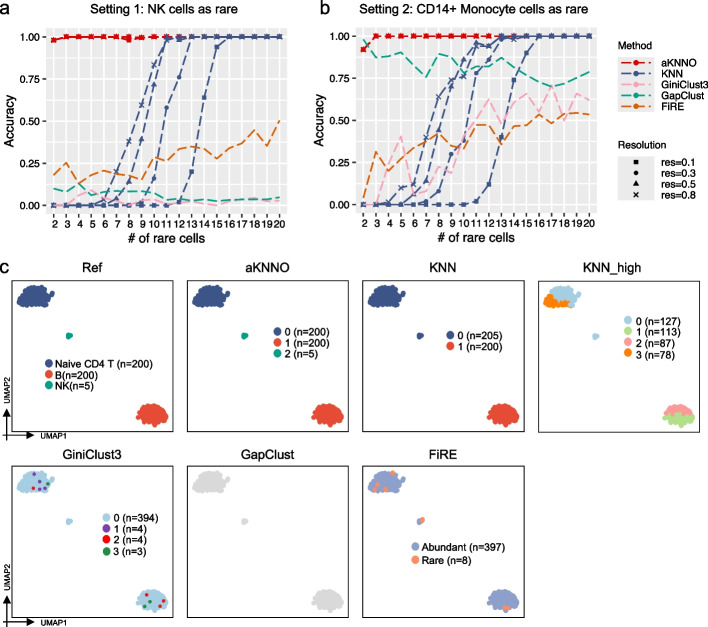
Application to simulated datasets generated from the PBMC3k dataset. Accuracy of aKNNO and KNN at different resolutions, GiniClust3, GapClust, and FiRE with the number of rare cells ranging from 2 to 20 in the first setting (**a**) and in the second setting (**b**). **c** The UMAP plots labeled by the ground truth (Ref), labeled by aKNNO clustering at a resolution of 0.1 (aKNNO), labeled by KNN clustering at a resolution of 0.1 (KNN), labeled by KNN clustering at a high resolution of 0.8 (KNN_high), labeled by GiniClust3, GapClust, and FiRE

We evaluated the performance by measuring both the accuracy of identifying rare cells (the percentage of rare cells correctly recognized) and the Adjusted Rand Index (ARI) against the true cell clusters. The clustering results were obtained at four different resolutions (*r* = 0.1, 0.3, 0.5, and 0.8). aKNNO demonstrated nearly perfect detection of rare cells (accuracy = 1) when the number of rare cells was greater than two in both settings. It achieved an accuracy exceeding 0.9 even when there were only two rare cells (Fig. 2a and b). Furthermore, aKNNO accurately grouped abundant cells and achieved nearly perfect agreement with the ground truth (average ARI > 0.995 in each scenario) (Additional file 1: Fig. S1). In comparison, KNN failed to detect rare cells entirely at every resolution when the number of rare cells was less than six in the first challenging setting (Fig. 2a). For example, with five rare NK cells (as illustrated in Fig. 2c), even at the lowest resolution of 0.1, aKNNO identified three clusters with 100% accuracy. KNN, however, grouped the five NK cells with naïve CD4 T cells, even at the highest resolution (KNN_high in Fig. 2c). As the number of rare cells increased, KNN showed improved performance in identifying them, yet its performance was sensitive to resolution (Additional file 1: Fig. S2). KNN exhibited higher accuracy with increasing resolution, whereas the accuracy of aKNNO remained stable across various resolutions (Additional file 1: Fig. S2). It is important to note that the higher accuracy of KNN at higher resolutions were achieved at the expense of overclustering of abundant cell types (an example was given in the Additional file 1: Fig. S3). In the second setting, KNN failed to detect rare cells when their number was less than five (Fig. 2b). Similarly, its performance was sensitive to resolution (Additional file 1: Fig. S2), and the improved performance at higher resolutions came at the cost of overclustering of abundant cell types. In summary, aKNNO demonstrates accurate identification of both abundant and rare cell types. Subcluster structures identified by aKNNO were supported by high Phiclust scores, derived from random matrix theory, which serves as a measure for identifying non-random substructure within cell clusters [22]. The high Phiclust scores demonstrated that aKNNO detected non-random substructures rather than statistical noise (Additional file 1: Fig. S4), that is, aKNNO identifies rare cells without overclustering, whereas KNN either fails to detect rare cells or does so at the expense of overclustering, especially when few rare cells are present.

We further compared aKNNO to three latest methods specifically designed or tailored to identify rare cells, GiniClust3 [18], FiRE [19], and GapClust [20]. GiniClust3 is an extension of GiniClust to identify both abundant and rare cell types, GapClust detects rare clusters, and FiRE quantifies rareness of each cell without clustering. All three methods exhibited poorer performance in identifying rare cells compared to aKNNO in both settings (Fig. 2a and b). In the first setting, FiRE had accuracy < = 0.5, while GapClust and GiniClust achieved accuracy < = 0.1 (Fig. 2a). As an example illustrated in Fig. 2c, FiRE identified two out of five rare NK cells, while both GapClust and GiniClust3 failed to detect any rare cells. Their performance improved notably in the less challenging second setting, particularly GapClust. GapClust performed slightly better than aKNNO when there were only two rare cells, but its performance significantly lagged behind aKNNO when the number of rare cells exceeded two (Fig. 2b).

### Application to single-cell transcriptomics data from human pancreas

**Fig. 3:**
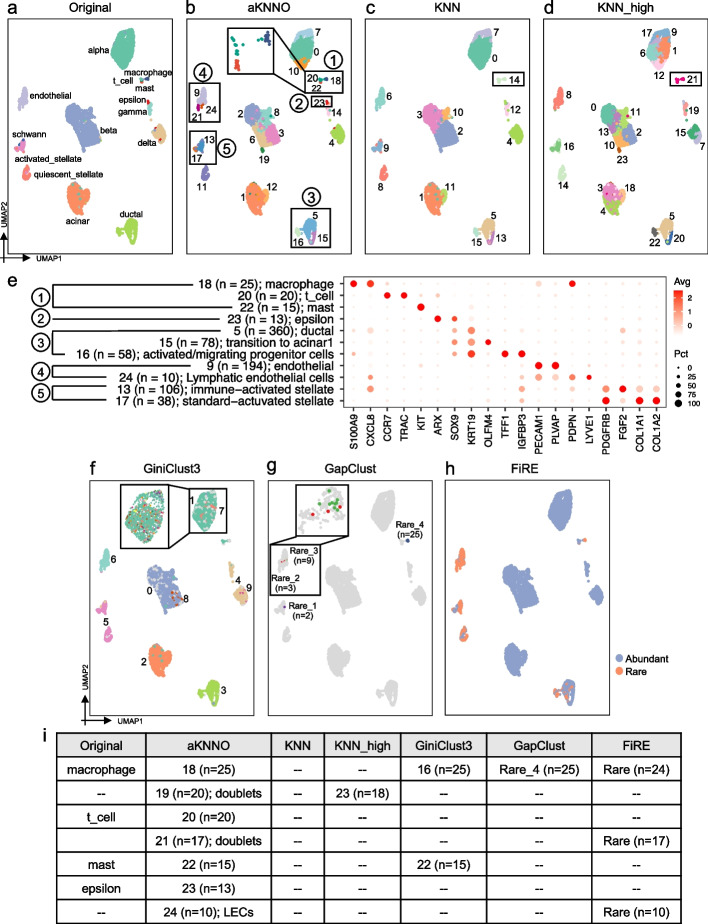
Application to single-cell RNAseq data from human pancreas. The UMAP plot labeled by the manual annotation from the original study (**a**), labeled by aKNNO clustering (**b**), KNN (**c**), KNN_high (**d**). **e** Dot plot of marker genes in clusters detected by aKNNO. **f** The UMAP plot labeled by the GiniClust3 result. Only the largest 10 clusters were shown since there were too many clusters. **g** The UMAP plot labeled by the GapClust result. **h** The UMAP plot labeled by the FiRE result. **i** A summary of rare clusters identified in the original study, aKNNO, KNN, KNN_high, GiniClust3, GapClust, and FiRE

We applied aKNNO to a single-cell RNAseq dataset from human pancreas by inDrops technology, involving 5542 cells and 14 cell populations manually annotated [23]. The 14 populations include three rare immune cell types, T, macrophage, and mast cells and one rare epsilon cell type (Fig. 3a). aKNNO identified 25 clusters in total (Fig. 3b). aKNNO identified all the manually annotated cell types except Schwann cells, including three rare immune cell types (clusters 18, 20, and 22 in Fig. 3b) and rare epsilon cells (cluster 23 in Fig. 3b, *n* = 13). It also returned refined subclusters for abundant cell types. Compared to one ductal cell type in the manual annotation, aKNNO found three ductal clusters, clusters 5 (*n* = 360), 15 (*n* = 78), and 16 (*n* = 58). The three clusters all expressed known ductal markers *KRT19* and *SOX9*, verifying their ductal identity. Compared to the abundant cluster 5, cluster 15 was specifically positive for *OLFM4*, and cluster 16 was high in *TFF1* and *IGFBP3* (Fig. 3e). These two clusters have been identified by a previous study on multipotent progenitor-like ductal cells, where *OLFM4* + ductal was named as “transition to acinar 1” and *TFF1* + *IGFBP3* + was labeled as “activated/migrating progenitor cells” [24]. Besides, aKNNO identified two endothelial clusters with high expression of *PECAM1* and *PLVAP* (clusters 9 and 24). Compared to cluster 9, the rare cluster 24 (*n* = 10) showed highly specific expression of *PDPN* and *LYVE1* (Fig. 3e), which are well-known markers for lymphatic endothelial cells (LEC) [25]. As another example, aKNNO identified two activated stellate clusters with high level of *PDGFRB* (clusters 13 and 17), the abundant cluster 13 (*n* = 106) with specific expression of *CXCL8* and *FGF2*, and the minor cluster 17 (*n* = 38) with specific expression of *COL1A1* and *COL1A2* (Fig. 3e). This division of activated stellate cells have been reported in the original literature by further analyzing stellate cells [23]. In addition to discovering true rare cell clusters, aKNNO also detected rare doublets, clusters 19 (*n* = 20) and 21 (*n* = 17). They had relatively higher number of genes and UMIs than other corresponding cell types and showed signatures from two different cell types, indicating they are doublets. Cluster 19 not only showed high expression of beta cell markers like *INS*, but also highly expressed acinar cell markers like *CPA1*. Cluster 21 had both high expression of endothelial (*PECAM1* and *PLVAP*) and stellate markers (*PDGFRB*) (Additional file 1: Fig. S5).

We compared aKNNO to KNN with default (*r* = 0.8) and high resolutions (*r* = 2, denoted as KNN_high). KNN discovered 16 clusters (Fig. 3c), while KNN_high identified 24 clusters in total (Fig. 3d). Surprisingly, KNN and KNN_high both failed to distinguish three rare immune clusters, T, macrophages, and mast cells (cluster 14 in the Fig. 3c and cluster 21 in the Fig. 3d), although they had very distinct features with high expression of *CCR7* and *TRAC* in T cells, *S100A9* and *CXCL8* in macrophages, and *KIT* in mast cells (Fig. 3e). KNN and KNN_high also missed the epsilon cells. KNN and KNN_high detected the two minor types of ductal cells but were unable to identify lymphatic endothelial cells, the two activated stellate cells, and doublets (Fig. 3c and d). Although KNN_high obtained 24 clusters, it overclustered abundant cell types rather than found rare clusters. With 25 clusters, in contrast, aKNNO found many true rare cell types and doublets without overclustering those abundant ones. There was high agreement between aKNNO and KNN in clustering abundant cells (ARI > 0.9), demonstrating that aKNNO is powerful in rare cell identification without scarifying its performance in abundant cells clustering.

We further compared rare cells identified by aKNNO, GiniClust3, FiRE, and GapClust. aKNNO identified seven rare clusters with less than or equal to 25 cells (clusters 18–24, *n* = 10 ~ 25, Fig. 3i), where cells in each cluster were densely located together in the UMAP embedding. They were either manually annotated in the original literature (T, mast, macrophage, and epsilon cells) or supported by well-known marker genes (LEC, and two types of doublets) (Fig. 3i), demonstrating they were true rare cells or clusters. In comparison, GiniClust3 identified 85 clusters in total (Fig. 3f). Although GiniClust3 obtained so many clusters, it misclassified even activated and quiescent stellate cells into one group (cluster 5 in the Fig. 3f) and three types of ductal cells into one cluster (cluster 2 in the Fig. 3f), indicating its poor performance in clustering abundant cell types. For the rare cell clusters, GiniClust3 identified mast, and macrophage, but missed epsilon cells, lymphatic endothelial cells, and two types of doublets (Fig. 3f and i). Most rare clusters identified by GiniClust3 scattered and mixed well with other abundant cells in the UMAP embedding and not detected by other methods, suggesting they were highly likely to be false rare cells (Fig. 3f). GapClust discovered four rare clusters, one of which is macrophage (*n* = 25, Rare_4 in the Fig. 3g). The other three rare clusters containing two, three, and nine cells, respectively, were highly likely to be false positives since they scattered and mixed with endothelial and activated stellate cells in the UMAP embedding (Fig. 3g). FiRE quantified 547 cells as being rare (Fig. 3h), which detected macrophage, lymphatic endothelial cells, and one type of doublets correctly but misidentified common endothelial cells and most of stellate cells as being rare (*n* > 150) and most of T cells and mast cells (*n* < 20) and all of epsilon cells (*n* = 13) as being common (Fig. 3h and i). In summary, aKNNO identified more true and less false rare cells than GiniClust3, GapClust, and FiRE.

### Application to single-cell transcriptomics from mouse brain

**Fig. 4:**
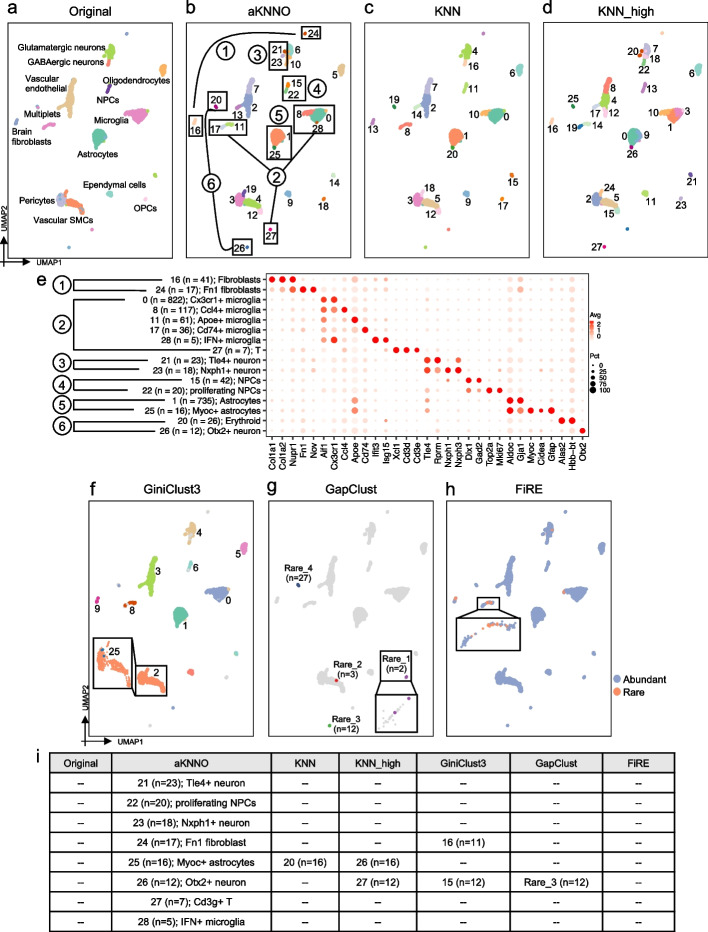
Application to single-cell RNAseq data from mouse brain. The UMAP plot labeled by the manual annotation from the original study (**a**), aKNNO (**b**), KNN (**c**), KNN_high (**d**). **e** The dot plot of marker genes in clusters detected by aKNNO. **f** The UMAP plot labeled by the GiniClust3 result. Only the largest 10 clusters were shown. **g** The UMAP plot labeled by the GapClust result. **h** The UMAP plot labeled by the FiRE result. **i** A summary of rare clusters identified in the original study, aKNNO, KNN, KNN_high, GiniClust3, GapClust, and FiRE

We applied aKNNO to a single-cell RNA-seq dataset from mouse brain by 10 × technology, involving 3985 cells and 12 cell types manually annotated [26]. However, the UMAP embedding showed far more than 12 clearly-separated clusters, suggesting the original annotation is rough and imprecise (Fig. 4a). For example, there are two separated groups annotated as brain fibroblasts and multiple distinct clusters all labeled as microglia (Fig. 4a). aKNNO detected 29 clusters in total, which perfectly identified those separated groups in the UMAP as different clusters (Fig. 4b). For the two distinct groups manually annotated as brain fibroblasts, aKNNO identified one as cluster 16 (*n* = 41), and the other as cluster 24 (*n* = 17) (Fig. 4b). Both clusters expressed known fibroblast markers *Col1a1*, *Col1a2* and *Nupr1* and cluster 24 also specifically expressed *Fn1* and *Nov*, which was known as Fn1 fibroblasts [27] (Fig. 4e). In comparison, KNN and KNN_high failed to distinguish these two types of fibroblasts (cluster 13 in the Fig. 4c and cluster 16 in the Fig. 4d). For the three distinct groups manually annotated as microglia, aKNNO detected six clusters (clusters 0, 8, 11, 17, 27, and 28 in the Fig. 4b). Five clusters had high expression of *Aif1*, a known marker of microglia, while the rare cluster 27 (*n* = 7) had low expression of *Aif1* but specifically expressed *Xcl1*, *Cd3d*, and *Cd3e* (Fig. 4e), suggesting its T cell identity. Each of the five types of microglia had specific gene expression signatures, cluster 0 (*n* = 822) with high *Cx3cr1*, cluster 8 (*n* = 117) with high *Ccl4*, cluster 11 with high *Apoe* (*n* = 61), rare cluster 17 (*n* = 36) with high *Cd74*, and rare cluster 28 (*n* = 5) with high *Ifit3* and *Isg15* (Fig. 4e). The microglia clusters 0, 8, 11, and 17 have different functions reported by previous studies [28, 29], and cluster 28 is highly enriched in IFN-response genes, suggesting it is a novel type of microglia. Among the five types of microglia and T cells, KNN only identified three and KNN_high found five types (Fig. 4c and d). Besides GABAergic and Glutamatergic neurons, aKNNO identified two more types of rare neurons (clusters 21 and 23 in the Fig. 4b, n = 23 and *n* = 18 respectively), where cluster 21 had specific expression of *Tle4* and *Rprm* and cluster 23 was positive for *Nxph1* and *Nxph3* (Fig. 4e). Those genes all play important roles in neurons [30], suggesting they are novel neuron types. KNN failed to identify both types of neurons, while KNN_high missed one type. In addition, AKNNO discovered two rare and distinct clusters (cluster 20, *n* = 26; cluster 26, *n* = 10 in the Fig. 4b), which were annotated as “multiplets” in the original annotation. Cluster 20 had high expression of *Alas2* and *Hbb-bt*, which are erythroid markers. Cluster 26 had specific expression of *Otx2*, which is known to regulate progenitor identity and neurogenesis in the midbrain [31, 32] (Fig. 4e). Besides, aKNNO and KNN were in high agreement on their clustering of abundant cells (ARI > 0.92), demonstrating aKNNO does not reduce their power at clustering abundant cells when it achieves great performance in identifying rare ones.

We further compared rare cells identified by aKNNO, GiniClust3, FiRE, and GapClust. aKNNO identified eight rare clusters with less than 25 cells (clusters 21–28, *n* = 5 ~ 23) (Fig. 4b and i). As discussed above, cluster 21 was *Tle4* + neuron, cluster 23 was *Nxph1* + neuron, cluster 24 was *Fn1* fibroblast, cluster 26 was Otx2 + neuron, cluster 27 was T cell, and cluster 28 was IFN + microglia. Cluster 22 was similar to cluster 10 with high expression of *Dlx1* and *Gad2*, which was manually annotated as neural progenitor cell (NPCs). However, cluster 22 expressed high *Top2a* and *Mki67* (Fig. 4e), suggesting it was proliferating NPCs. Cluster 25 and cluster 1 both had high expression of known astrocytes markers, such as *Aldoc* and *Gja1*. Cluster 25 had specific expression of *Myoc*, *Cidea*, and *Gfap* (Fig. 4e), which has been reported as a new type of Astrocytes [33]. In comparison, GiniCluster3 identified 27 clusters in total (Fig. 4f). For abundant cell types, it misidentified pericytes and vascular SMCs as one group (cluster 2 in the Fig. 4f), indicating its inefficiency in large cluster detection. Among the eight rare cell types identified aKNNO, GiniClust3 only identified Fn1 fibroblast and Otx2 + neuron (Fig. 4i). Cells in other rare clusters by GiniClust3 scattered in the UMAP embedding and mixed with other abundant cells, indicating they are not true rare cells (Fig. 4f). GapClust obtained four rare clusters, containing 2, 3, 12, and 27 cells, respectively (Fig. 4g). The cluster with 12 cells (Rare_3 in the Fig. 4g) corresponded to cluster 26 of aKNNO, which is Otx2 + neuron. The cluster with 27 cells (Rare_4 in the Fig. 4g) matched cluster 20 in the aKNNO, which was not that rare compared to the eight rare clusters. The other two rare clusters mixed with abundant cells in the UMAP embedding (Fig. 4g), suggesting they are not real rare. FiRE only found 56 rare cells (Fig. 4h), most of which mixed with microglia and fibroblasts. It failed to detect any of the eight rare clusters in aKNNO (Fig. 4i). Consistent with the results from human pancreas, aKNNO achieved higher sensitivity and specificity than other methods in identifying rare cells.

### Application to spatial transcriptomics data from mouse posterior brain

Spatial transcriptomics map out organizational structures of cells along with their transcriptomics profiles, providing powerful tools for understanding spatial and functional arrangement of tissues [34].

**Fig. 5:**
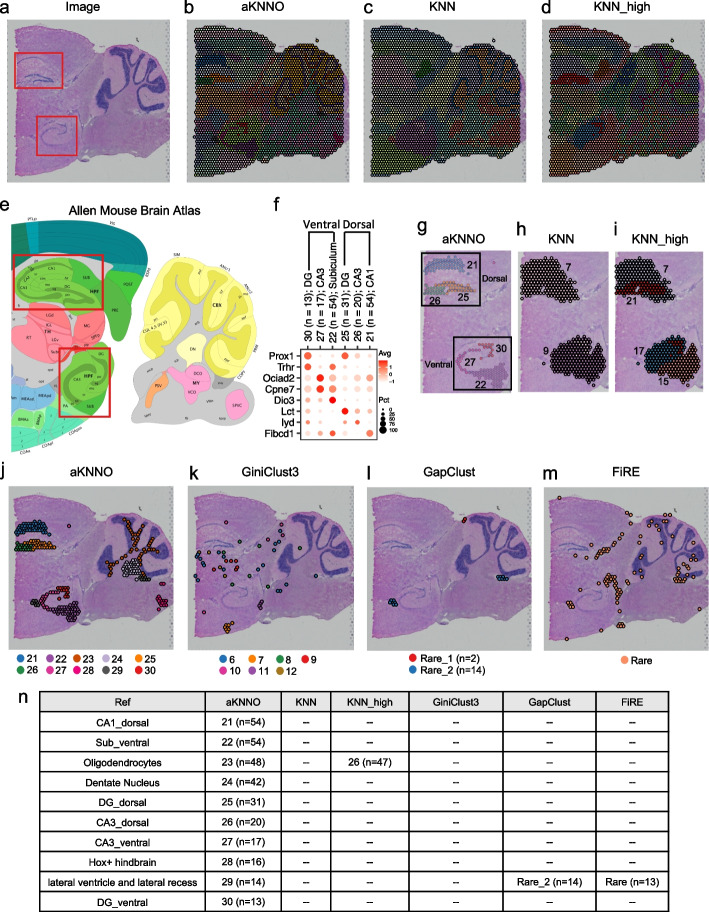
Application to 10 × Visium spatial transcriptomics data from mouse posterior brain. **a** H&E image. The spatial plot annotated by aKNNO (**b**), KNN (**c**), and KNN_high (**d**). **e** Allen Brain Institute reference atlas diagram. **f** Dot plot of marker genes in the dorsal and ventral hippocampus structures. The spatial plot focusing on dorsal and ventral hippocampus structures annotated by the six cell clusters identified by aKNNO (**g**), two cell clusters detected by KNN (**h**), three cell clusters by KNN_high (**i**). The spatial plot of rare cell clusters detected by aKNNO (**j**), GiniClust3 (**k**), GapClust (**l**), and FiRE (**m**). **n** A summary of rare clusters identified by aKNNO, KNN, KNN_high, GiniClust3, GapClust, and FiRE

We applied aKNNO to a 10 × Visium dataset generated from mouse sagittal posterior brain, which has complicated tissue structures. We used the brain anatomical reference annotations from the Allen Mouse Brain Atlas and H&E image as the ground truth (Fig. 5a and e). aKNNO identified 31 clusters from the 3355 spots, which stereotyped anatomical structures precisely (Fig. 5b). For example, the hippocampal system consists of the dentate gyrus (DG), cornu ammonis (CA) fields that are subdivided into four regions (CA1–CA4), and the subiculum [35]. The brain anatomical reference annotations and the H&E image of the data show dorsal and ventral hippocampus, where the dorsal contains DG, CA3, and CA1, and the ventral includes DG, CA3, and subiculum (highlighted in red boxes in the Fig. 5a and e). Previous transcriptomics studies on neuronal classes of the hippocampus found not only cell-class but also region-specific expression profiles, indicating heterogeneous populations along the dorsal-ventral axis [36]. aKNNO discovered six clusters successfully with known cell- and region-specific expression (clusters 21, 22, 25, 26, 27, and 30 in the Fig. 5g), which mapped to the six anatomical patterns precisely. In the ventral hippocampus, cluster 30 (*n* = 13) aligned exclusively to DG and had high expression of known DG-specific (*Prox1*) and ventral-specific genes (*Trhr*) [36] (Fig. 5f). Cluster 27 (*n* = 17) depicted CA3 with CA3-specific expression of *Ociad2* and *Cpne7* [36], and cluster 22 corresponded to subiculum with known cell-class and region-specific expression of *Dio3* [37] (Fig. 5f). In the dorsal region, cluster 25 (*n* = 31) lined up with DG with DG-specific gene *Prox1* and dorsal-specific gene *Lct* [36], cluster 26 (*n* = 20) mapped to CA3 with high CA3_dorsal-specific expression of *Iyd* [36], and cluster 21 (*n* = 54) matched CA1 with high expression of *Fibcd1* [36] (Fig. 5f). In comparison, KNN and KNN_high discovered 15 and 28 clusters, respectively, which showed poorer performance in defining anatomical patterns than aKNNO (Fig. 5c and d). For example, KNN and KNN_high failed to distinguish the six hippocampus subpopulations. KNN only found two clusters, where it misclassified DG_ventral and the whole dorsal hippocampus into one cluster, and it miscategorized CA3_ventral and subiculum into one and also masked them into the surrounding region (Fig. 5h). KNN_high detected four clusters, where it misclassified DG_ventral, CA3_dorsal, and DG_dorsal into one cluster, and it failed to distinct CA1_dorsal, CA3_ventral and subiculum from their surrounding regions (Fig. 5i).

The ten rare clusters identified by aKNNO (clusters 21–30 in the Fig. 5j, n < 55) all aligned exclusively to a specific brain region with functional meaning, including CA1_dorsal (cluster 21, *n* = 54), subiculum_ventral (cluster 22, *n* = 54), oligodendrocytes in the white matter (cluster 23, *n* = 48), dentate nucleus (cluster 24, *n* = 42), DG_dorsal (cluster 25, *n* = 31), CA3_dorsal (cluster 26, *n* = 20), CA3_ventral (cluster 27, *n* = 17), Hox gene-enriched hindbrain (cluster 28, *n* = 16), lateral ventricle and lateral recess (cluster 29, *n* = 14), and DG_ventral (cluster 30, *n* = 13) (Fig. 5e, j and n). In comparison, GiniClust3 detected only 13 clusters, which agreed poorly with the Allen Brain Institute reference atlas diagram (Additional file 1: Fig. S6). For example, multiple cortical layers were misclassified into one cluster and none of the hippocampus structure were identified correctly (Additional file 1: Fig. S6). The rare clusters (clusters 6–12 in the Fig. 5k with *n* < 55) scattered randomly in the spatial region and did not map to specific anatomical regions, suggesting that they were highly likely to be false. None of the ten rare clusters in aKNNO were detected by GiniCluster3 (Fig. 5n). GapClust only identified two rare clusters (Fig. 5l). One cluster containing 14 cells aligned to lateral ventricle and lateral recess region (cluster 29 in the aKNNO), while the other cluster with two cells was identified to be common by other methods. FiRE identified 105 rare cells (Fig. 5m). Among them, 13 cells were lined up with lateral ventricle and lateral recess region (cluster 29 in the aKNNO). Sixty-six cells were smooth muscle cells with high level of *Ogn* and *Prdm6*, which were also identified by aKNNO (Additional file 1: Fig. S7, *n* = 105) and were not that rare compared to the ten rare clusters. Other cells scattered in the brain region and might not be true rare cells. In summary, the ten rare clusters identified by aKNNO stereotyped subtle anatomical structures, whereas GapClust and FiRE only recovered the lateral ventricle and lateral recess structure (Fig. 5n).

**Fig. 6:**
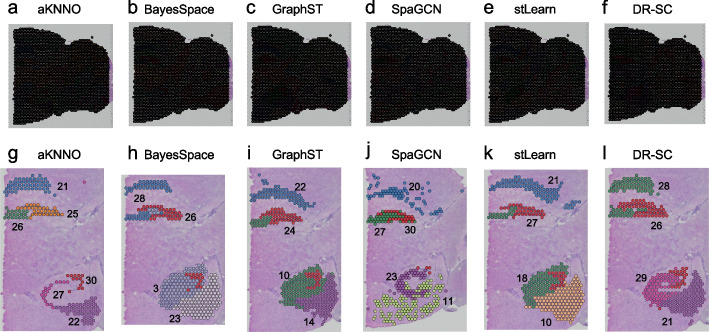
Comparison between aKNNO, BayesSpace, GraphST, SpaGCN, stLearn, and DR-SC on the 10 × Visium spatial transcriptomics data from mouse posterior brain. The spatial pot annotated by aKNNO (**a**), BayesSpace (**b**), GraphST (**c**), SpaGCN (**d**), stLearn (**e**), and DR-SC (**f**). Detailed view of clustering in hippocampus structures in aKNNO (**g**), BayesSpace (**h**), GraphST (**i**), SpaGCN (**j**), stLearn (**k**), and DR-SC (**l**)

Recently, several integrative approaches combining expression, spatial location, and histology images have been developed to improve the performance of spatial transcriptomics clustering [38–42]. We compared aKNNO with five integrative approaches, stLearn [39], SpaGCN [38], GraphST [40], BayesSpace [41], and DR-SC[42]. We set their number of clusters to 31, matching the cluster number of aKNNO (Fig. 6a–f). Using the fine-grained hippocampus structures as an example, aKNNO stereotyped six anatomical patterns precisely (Fig. 6g). In contrast, all the five integrative approaches failed to resolve the six patterns. Both BayesSpace and DR-SC misclassified CA3_dorsal and CA1_dorsal into one (cluster 28 in Fig. 6h and l) as well as DG_dorsal and DG_ventral into one (cluster 26 in Fig. 6h and l). However, DR-SC successfully identified CA3_ventral and subiculum (clusters 29 and 21 in Fig. 6l), while BayesSpace failed to distinguish them from their surrounding regions (clusters 3 and 23 in Fig. 6h). GraphST identified CA1_dorsal (cluster 22 in Fig. 6i) but misrecognized DG_dorsal and DG_ventral into one (cluster 24 in Fig. 6i) and CA3_dorsal and CA3_ventral into one (Cluster 10 in Fig. 6i). SpaGCN successfully identified CA3_dorsal (cluster 27 in Fig. 6j) and CA3_ventral (cluster 23 in Fig. 6j) but misclassified DG_dorsal and DG_ventral into one (cluster 30 in Fig. 6j) and also lost the subiculum structure. stLearn, similar to GraphST, detected CA1_dorsal (cluster 21 in Fig. 6k) but failed to distinguish between CA3_dorsal and CA3_ventral (cluster 18 in Fig. 6k) and between DG_dorsal and DG_ventral (cluster 27 in the Fig. 6k). The comparison demonstrated that aKNNO, using gene expression alone, resolved tissue structures more accurately than those integrative approaches.

## Discussion

The accurate detection of abundant and rare clusters simultaneously is crucial for characterizing cellular heterogeneity in single-cell and spatial transcriptomics analysis. Here, we presented aKNNO, an adaptive *k*-nearest neighbor graph with optimization for the community-detection-based clustering. Compared to traditional *k*NN specifying a universal *k* for all cells, aKNNO chooses *k* adaptively for each cell based on its local distance distribution. The adaptive strategy assigns a small *k* for rare cells and a large *k* for abundant cells, enabling to capture the inherent cellular structure accurately. aKNNO has been extensively evaluated on 38 simulated scenarios and 20 single-cell and spatial transcriptomics data from different species, tissues, and technologies. Additional analysis on mouse intestine from 10x [43], mouse habenula from inDrops [44], organoids from CEL-seq [8], spatial main olfactory bulb, spatial coronal posterior brain, and 12 pan-cancer datasets [45, 46] were included in the Additional file 1: Sections S1-S6. The results consistently demonstrated that aKNNO outperformed traditional *k*NN-based approach in identifying abundant and rare cell types in a single run. aKNNO identifies rare cells without overclustering abundant cells. In both simulated and real datasets, aKNNO did not overcluster those *k*NN-based clusters with *∅*_*clust*_ = 0*.* The substructures identified by aKNNO demonstrate both high Phiclust scores and distinct well-known markers, highlighting their biological relevance over mere statistical noise (Additional file 1: Figs. S3, S4, and S8). It is important to note that aKNNO aims to construct a graph that accurately capture inherent cellular structure, rather than pinpointing the optimal clustering resolution. In fact, aKNNO can be clustered at any resolution. Due to Phiclust scores being sensitive to technical and biological noise, high Phiclust scores, coupled with the presence of distinct, meaningful markers, might reliably indicate the existence of true subclusters. aKNNO was also far more superior than those methods specifically designed or tailored for rare cells detection in terms of both sensitivity and specificity.

aKNNO provides an optimization step to tune $\delta$, a hyperparameter controlling the sensitivity to local distance change. High sensitivity to local distance change would result in many localized subclusters and lead to overclustering, while low sensitivity would lose the adaptive ability and reduce to the traditional *k*NN. aKNNO uses grid search to find the optimal $\delta$ for each dataset. We compared the performance between the optimal and the default $\delta$ (− 0.5). We found the optimal $\delta$ was different across datasets. aKNNO using the optimal $\delta$ was able to find more true rare cell types than the approach using the default $\delta$ without optimization (Additional file 1: Figs. S9-S12). That is, the optimization step did help balance between sensitivity and specificity in rare cells identification. aKNNO is less sensitive to clustering resolution than *k*NN-based approaches thanks to its ability to capture the inherent cellular structure more accurately (Additional file 1: Fig. S2). Furthermore, the performance of aKNNO is unaffected by the parameter *K*_max_, representing the maximum number of nearest neighbors. We conducted a sensitivity analysis for the parameter *K*_max_ in simulation studies, demonstrating that the performance of aKNNO remains consistent across varying values of *K*_max_ (ranging from 10 to 50) when the number of rare cells ranges from 2 to 10 (Additional file 1: Fig. S13).

aKNNO builds a nearest neighbor graph, which can be seamlessly incorporated into existing analysis pipelines (see tutorials in the GitHub) and also used for any graph-based clustering approaches, such as Louvain clustering, spectral clustering, Leiden clustering, and Minimal Spanning Tree. aKNNO not only identified true rare clusters but also found doublets and empty droplets. For example, aKNNO found two types of doublets in the human pancreas and one cluster with only two empty droplets in the mouse habenula dataset, that is, aKNNO can be used to remove poor quality cells that are not eliminated completely in the preprocessing step. Although an adaptive strategy is included to build the NN-graph, the computational burden added is neglectable compared to the traditional *k*NN-based approach. aKNNO is comparable to the traditional *k*NN-based approach and significantly more efficient than GapClust, GiniClust3, and FiRE in terms of run time and memory usage (Additional file 1: Fig. S14).

Spatial transcriptomics is an emerging technology that provides a roadmap of transcriptional activity within tissue sections. To better decipher domains or cell types that are spatially coherent in both gene expression and histology, a number of integrative approaches to combine gene expression, spatial location, histology, and H&E image have been developed [38–42]. Integrating multi-modal information are expected to define cell types or domains accurately than using gene expression alone. Surprisingly, aKNNO is able to stereotype anatomical cell types precisely using gene expression alone. For example, the six clusters identified by aKNNO aligned almost perfectly with the six dorsal and ventral hippocampus structures (DG_dorsal, DG_ventral, CA3_dorsal, CA3_ventral, CA1_dorsal, and subiculum_ventral). Each common and rare cluster detected by aKNNO mapped to specific regions in the Allen Brain Institute reference atlas diagram, suggesting they are functionally meaningful. Some clusters or spatial regions were even not identified by those integrative approaches (Fig. 6 and Additional file 1: Sections S4-S5). The results suggest that gene expression alone is enough to define spatial cell types or domains when the appropriate strategy is used.

## Conclusions

We proposed aKNNO, a novel clustering method to identify abundant and rare cell types simultaneously for single-cell and spatial transcriptomics data. aKNNO benefits from choosing *k* adaptively for each cell based on its local distance distribution, which captures inherent cellular structure accurately. Without sacrificing performance for clustering abundant cell types, aKNNO discovered known and novel rare cell types that those typical and even specifically tailored methods failed to detect. Notably, aKNNO using transcriptome alone stereotyped fine-grained anatomical structures more precisely than those spatially and histology informed approaches. aKNNO provides a powerful and accurate way in unsupervised clustering of single-cell and spatial transcriptomics data.

## Methods

### Single-cell and spatial transcriptomics data processing

Single-cell RNAseq datasets were filtered and processed by the Seurat package [15]. Specifically, cells expressing less than 200 genes and genes expressed in less than three cells were excluded. Data were normalized to the 10,000 UMI, and the top 2000 highly variable genes were selected by the vst method. Principal component analysis (PCA) was used to reduce dimension and UMAP embedding was computed for visualization.

Spatial transcriptomics data were processed by the Seurat package [47]. Particularly, data were normalized and the top 3000 highly variable genes were selected using SCTtransform [48]. Genes expressed in less than 10% of spots in the mouse sagittal posterior brain were filtered out, following preprocessing steps in previous studies [38, 49]. Principal component analysis (PCA) was used to reduce dimension and UMAP embedding was computed for visualization.

### aKNNO

aKNNO includes five steps: (1) calculate the distances within the *K*_*max*_ nearest neighbors for all cells; (2) choose the actual number of the true nearest neighbors *k* adaptively for each cell to construct an adaptive *k*NN graph; (3) build the shared nearest neighbor graph; (4) Louvain clustering on the shared neighbor graph; (5) repeat steps (2)–(4) for optimization (Fig. 1).

1. Calculate the distances within the *K*_*max*_ nearest neighbors for all cells; the top PCs (default: 50) were used to calculate the distances between cells. Given a maximum number of the nearest neighbors *K*_*max*_, RANN package is used to find the *K*_*max*_ nearest neighbors of all cells and calculate the distances
2. Construct an adaptive *k*NN graph by choosing* k* adaptively for each cell; for each cell *i*, the distance to its *K*_*max*_ nearest neighbors are sorted in an ascending order (*d*_*i*1_ < *d*_*i*2_ < …*d*_*iK*max_) and *k* is chosen based on the *K*_*max*_ distance distribution [50]. Several algorithms have been proposed to find adaptive neighbors [51–54]. We used the algorithm described in Cai et al. [50], which does not require the regularization term and thus be more computational efficient

Assuming there are *n* cells in total (1,2,…*n*), for the cell *i*, the cell *j* can be connected to *i* as a neighbor with probability S_*ij*_. Intuitively, a smaller distance should be assigned a larger probability. Cai et al. (Cai et al. [50]) computes the probability by minimizing the following objective function:

1 $$min\sum\nolimits_{i=1}^{n}\sum\nolimits_{j=1}^{n}\sum\nolimits_{m=0}^{\infty }({d}_{ij}{s}_{ij}^{m})$$

$$s.t.{\forall }_{i}, 0\le {s}_{ij}\le 1, {s}_{i}^{T}1=1$$

where *m* is the number of power, and ${d}_{ij}$ is the distance between the cell *i* and *j*. The standard Karush–Kuhn–Tucker conditions are used to solve the Eq. (1). The optimal solution is:

$${s}_{ij}=\left\{\begin{array}{c}1, if \,{d}_{ij} <{(\frac{{\sum }_{l=1}^{{K}_{max}}\sqrt{{d}_{il}}}{{K}_{max}-1-\delta })}^{2} \\ 0, otherwise\end{array}\right.$$

where $\delta$ is a hyperparameter to control the sensitivity to the local distance change ($\delta \le 0)$. ${s}_{ij}=1$ indicates there is a connection between *i* and *j*, while ${s}_{ij}=0$ means no connection. Therefore, for the *K*_*max*_ nearest neighbors of the cell *i (d*_*i*1_ < *d*_*i*2_ < …*d*_*i*_*K*_*max*_),

$$k_i=\left\{\begin{array}{c}K_{max},if\;d_{iK_{max}}<{(\frac{\sum_{l=1}^{K_{max}}\sqrt{d_{il}}}{K_{max}-1-\delta})}^2\\j,if\;d_{ij}<{(\frac{\sum_{l=1}^{K_{max}}\sqrt{d_{il}}}{K_{max}-1-\delta})}^2and\;d_{i(j+1)}\geq\left(\frac{\sum_{l=1}^{K_{max}}\sqrt{d_{il}}}{K_{max}-1-\delta}\right)^2,j=\text{1,2},\dots K_{max}-1\end{array}\right.$$

For example, the true nearest neighbor *k* for the cell *i* is set to 3 automatically (*k*_*i*_ = 3) if $d_{i3}<{(\frac{\sum_{l=1}^{K_{max}}\sqrt{d_{il}}}{K_{max}-1-\delta})}^2and\;d_{i4}\geq{(\frac{\sum_{l=1}^{K_{max}}\sqrt{d_{il}}}{K_{max}-1-\delta})}^2$. In this way, aKNNO chooses the *k* adaptively based on the local distance distribution for each cell.

3) Build the shared nearest neighbor graph; The adaptive *k*NN graph is reweighted based on the shared nearest neighbors of each pair of cells, which make it more robust to outliers and noise. The Jaccard similarity is used to build the shared nearest neighbor graph.
4) Louvain clustering on the shared neighbor graph; the Louvain clustering in the Seurat package is used to group cells on the shared nearest neighbor graph.
5) Repeat steps (2)–(4) for optimization. $\delta$ is a hyperparameter to control the sensitivity to the local distance change (default = − 0.5). A too negative $\delta$ would make aKNNO very sensitive to local distance change and lead to overclustering, while aKNNO would lose the adaptive ability if $\delta$ is not negative enough. To find the optimal $\delta$, aKNNO employs a grid search to find the $\delta$ that balances between the sensitivity and specificity. aKNNO decreases $\delta$ in each repeat and choose the $\delta$ before there is a rapid increase in the number of communities detected, which suggests overclustering (Fig. 1).

### Comparison with other methods

We compared aKNNO with the conventional *k*NN-based clustering method in the Seurat package (denoted as KNN). aKNNO identified clusters at the default solution (*r* = 0.8), while KNN found clusters at the default resolution (*r* = 0.8) and a high resolution (*r* = 2, denoted as KNN_high). To make a fair comparison, aKNNO and KNN processed the data exactly the same way, including the highly variable genes selection, the number of PCs to calculate the distance, the method to find the nearest neighbors, and Louvain clustering. The only difference between aKNNO and KNN is the graph used for clustering. aKNNO generates clustering from the adaptive *k*NN graph, while KNN from the traditional *k*NN graph.

We also compared aKNNO with three methods specifically designed or tailored to identify rare cell types, GiniClust3 [18], GapClust [20], and FiRE [19]. GiniClust3 is an extension of GiniClust, which discovers rare cells based on genes with high Gini index. FiRE uses the Sketching technique to assign a rareness score to each cell [19]. GapClust captures the abrupt local distances change to find rare cell clusters [20]. GiniClust3 identifies abundant and rare clusters simultaneously, GapClust finds rare clusters only, and FiRE quantifies rareness of each cell without clustering. Default parameters were used to run GiniClust3, GapClust, and FiRE. GapClust requires a normalized expression matrix as the input; therefore, the data were normalized to 10,000 UMI without log-transformation for GapClust.

In the spatial transcriptomics data, we further compared aKNNO with five integrative approaches, BayesSpace [41], GraphST [40], SpaGCN [38], stLearn [39], and DR-SC[42]. BayesSpace implements a full Bayesian model that uses the information from spatial neighborhoods for resolution enhancement for spatial clustering. stLearn first normalizes gene expression by distance measures on morphological similarity and neighborhood smoothing and then clustering cell types from the normalized expression profiles. SpaGCN uses a graph convolutional network that integrate gene expression, spatial location, and histology images. GraphST is a graph self-supervised contrastive learning method that combines spatial location and expression. DR-SC simultaneously performs dimension reduction and spatial clustering within a unified framework, which encourages spatial smoothness based on a latent hidden Markov random field. Default parameters were used to run BayesSpace, GraphST, SpaGCN, stLearn, and DR-SC. The number of clusters was set to the same as aKNNO.

### Simulation studies

We generated simulated datasets by sampling four cell types from the PBMC3k dataset: B cells, naïve CD4 T cells, NK, and CD14 + monocytes. We created two settings. In one setting, the rare cell type was similar to one of the abundant cell types, i.e., rare NK but abundant B (*n* = 200) and naïve CD4 T (*n* = 200) cells. In the other setting, the rare cell type was distinct from both abundant cell types, i.e., rare CD14 + monocytes but abundant B (*n* = 200) and naïve CD4 T cells (*n* = 200). In each setting, we simulated 19 scenarios with the number of rare cells ranging from 2 to 20. In each scenario, 50 simulated datasets were generated by random sampling the four cell types from the PBMC3k dataset, which was downloaded from https://cf.10xgenomics.com/samples/cell/pbmc3k/pbmc3k_filtered_gene_bc_matrices.tar.gz.

### Supplementary Information

Additional file 1. Supplementary information, includes supplementary figures and supplementary sections for other applications of aKNNO.

Additional file 2. Review history.

## Data Availability

The R package aKNNO, together with tutorials for reproducing the analysis results presented in the study, is freely available on GitHub (https://github.com/liuqivandy/aKNNO) [55] and Zenodo [56]. The source code is released under the GNU General Public License v3.0. The single-cell RNAseq data for human pancreas, mouse brain, mouse intestine, mouse habenula, and intestinal organoids are available at the Gene Expression Omnibus (GEO) under accession numbers GSE84133 [23, 57], GSM3580745 [26, 58], GSM4521364 [59], GSM4411753 [44, 60], and GSE62270 [8, 61–63], respectively. The 10x Visium data for mouse coronal and sagittal posterior brain [64] and main olfactory bulb are downloaded from the 10x Genomics website (https://www.10xgenomics.com/resources/datasets). The single-cell RNAseq dataset of the melanoma is obtained from GEO under accession number GSE72056 [45, 65], and the other 11 cancer datasets were downloaded from the database Tumor Immune Single-cell Hub 2 (TISCH2) [46, 66–73]. Lymphatic endothelial cells (LEC) were identified in 12 datasets [74–79].
