# Supplementary material for: aKNNO: single-cell and spatial transcriptomics clustering with an optimized adaptive k-nearest neighbor graph
Source: Genome Biol. 2024 Aug 1;25:203. doi: 10.1186/s13059-024-03339-y (PMC11293182; doi:10.1186/s13059-024-03339-y)
Supplement: Supplementary file 1 — Additional file 1. Supplementary information, includes supplementary figures and supplementary sections for other applications of aKNNO. [file 13059_2024_3339_MOESM1_ESM.docx]

**Supplementary Information**

**aKNNO: single-cell and spatial transcriptomics clustering with an optimized adaptive k-nearest neighbor graph**

Jia Li^1,2^, Yu Shyr^1,2*^, Qi Liu^1,2*^

^1^Department of Biostatistics, Vanderbilt University Medical Center, Nashville, TN, 37203, USA

^2^Center for Quantitative Sciences, Vanderbilt University Medical Center, Nashville, TN, 37203, USA

*To whom correspondence should be addressed: Tel: +1 615 322 6618; Fax: +1 615 936 2602; Email: [qi.liu@vumc.org](mailto:qi.liu@vumc.org)

*Correspondence may also be addressed to: Tel: +1 615 936 0682; Fax: +1 615 936 2602; Email: [yu.shyr@vumc.org](mailto:yu.shyr@vumc.org)

**Fig. S1.** The highest average adjusted rand index of aKNNO (red) and KNN (blue) with the ground truth in the first setting (a) and in the second setting (b). The highest adjusted rand index across different resolutions was selected and the average ARI across 50 datasets was calculated.

**Fig. S2.** The accuracy of aKNNO and KNN at different resolutions with the number of rare cells from 2 to 20 in the two different settings.

**Fig. S3.** The performance of aKNNO, KNN, and KNN_high on a simulated dataset with 12 rare NK cells, 200 naïve CD4 T cells, and 200 B cells.

**Fig. S4.** Phiclust scores on KNN clustering results for a simulated dataset in the first setting with 5 rare NK cells, 200 naïve CD4 T cells, and 200 B cells (a); for a simulated dataset in the second setting with 5 rare CD14+ monocytes, 200 naïve CD4 T cells, and 200 B cells (b). KNN incorrectly grouped rare NK or CD14+ monocytes with naïve CD4 T cells (labeled as cluster 0 in the figure). Cluster 0 exhibits high phiclust scores ($\emptyset$=0.77 and $\emptyset$=0.85 in the two settings, respectively), indicating the presence of non-random substructures and the necessity for further subclustering. In contrast, Cluster 1 (B cells) has a $\emptyset$=0, suggesting the absence of non-random substructure. As anticipated, aKNNO successfully subdivided cluster 0 into two subclusters without overclustering cluster 1.

KNN

aKNNO

KNN

aKNNO


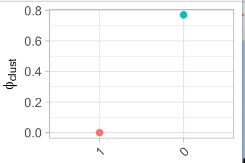


aKNNO: 0 (Naïve CD4 T) + 2 (NK)

KNN:

KNN:

𝛷=0.77


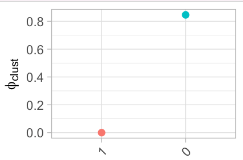


aKNNO: 1(B)

aKNNO: 0 (Naïve CD4 T) + 2(CD14+ Mono)

KNN:

KNN:

𝛷=0.85

aKNNO: 1(B)

a

b

**Fig. S5.** The distribution of UMI counts, the number of genes, gene signatures in the cluster 19 and the cluster 21 identified by aKNNO in the human pancreas data. The cluster 19 (a) and the cluster 21 (b) had both higher UMI counts and number of genes than other corresponding clusters. (c) The cluster 19 expressed high beta (*INS*) and acinar markers (*CPA1*). The cluster 21 had both high expression of endothelial (*PECAM1* and *PLVAP*) and stellate markers (*PDGFRB* and *RGS5*).

**Fig. S6.** GiniCluster3 clustering on the 10x Visium mouse posterior brain

**Fig. S7.** (a) The cluster 16 identified by aKNNO. (b) Spatial expression plots of *Ogn* and *Prdm6* in the 10x Visium mouse posterior brain data.

**Fig. S8.** Phiclust scores on clusters identified by KNN in mouse brain(a), mouse intestinal epithelium(b), and mouse intestinal organoid (c). aKNNO did not overcluster those kNN-based clusters with zero phiclust scores. In contrast, it identified subclusters in those kNN-based clusters with relatively high phiclust scores.

**Fig. S9.** Performance of aKNNO and aKNNO without optimization at the default delta of -0.5 on the human pancreas data. (a) The number of communities and singletons detected at different delta values. The optimal delta of -0.8 (dash line) was chosen automatically. (b) Clustering of aKNNO. (c) Clustering of aKNNO at delta of -0.5 without optimization.

**Fig. S10.** Performance of aKNNO and aKNNO without optimization at the default delta of -0.5 on the mouse brain data. (a) The number of communities and singletons detected at different delta values. The optimal delta of -0.8 was chosen automatically. (b) Clustering of aKNNO. (c) Clustering of aKNNO at delta of -0.5 without optimization.

**Fig. S11.** Performance of aKNNO and aKNNO without optimization at the default delta of -0.5 on the mouse intestine data. (a) The number of communities and singletons detected at different delta values. The optimal delta of -0.9 was chosen automatically. (b) Clustering of aKNNO. (c) Clustering of aKNNO at delta of -0.5 without optimization.

**Fig. S12.** Performance of aKNNO and aKNNO without optimization at the default delta of -0.5 for the 10x Visium mouse posterior brain data. (a) The number of communities and singletons detected at different delta values. The optimal delta of -0.9 was chosen automatically. (b) Results of aKNNO. (c) Results of aKNNO at delta of -0.5 without optimization.

**Fig. S13.** The accuracy of aKNNO at different K_max with the number of rare cells from 2 to 10 in the two simulation datasets generated by two different settings.

**Fig. S14.** a) Runtime of aKNNO, KNN, GiniClust3, Gapclust, and FiRE as the number of cells increases. b) Maximum memory usage of aKNNO, KNN, GiniClust3, Gapclust, and FiRE for 10,000 cells.

**Section S1:Application to single-cell RNA-seq from mouse intestinal**

We analyzed one single-cell RNA-seq dataset from mouse intestine containing 559 cells [1]. aKNNO uncovered 10 clusters, which included all known cell types in the intestine epithelium (Additional file 1: Fig. S15a), including stem (cluster 3, n=65), TA (Transit Amplifying) (cluster 1, n=95), intermediate enterocytes (clusters 0 and 7), mature enterocytes (cluster 4, n=62), goblets (cluster 2, n=85), k cells (cluster 5, n=48), enteroendocrine (cluster 6, n=28), tuft (cluster 8,n=5), and Paneth cells (cluster 9, n=3). Their identities were verified by known markers, such as high level of *Ascl2* and *Lgr5* in stem, *Top2a* and *Mki67* in TA, *Fabp6* and *Apoa1* in mature enterocytes, *Muc2* and *Clca1* in goblets, *Gip* in k cells, *Chga* and *Chgb* in enteroendocrine, *Hck* and *Lrmp* in Tuft, and *Defa21* and *Defa22* in Paneth cells (Additional file 1: Fig. S15d). In comparison, KNN found five clusters and KNN_high detected nine clusters (Additional file 1: Figs. S15b and S15c). Neither KNN nor KNN_high identified those rare cell types successfully, including k cells, enteroendocrine, tuft, and paneth cells. They both failed to separate k cells and enteroendocrine (Additional file 1: Figs. S15b and S15c). In KNN and KNN_high, Paneth cells were hidden by goblets, while tuft cells were misclassified into enterocytes (Additional file 1: Figs. S15b and S15c).

GiniClust3 identified nine clusters in total, among of which six rare groups had less than 10 cells (Additional file 1: Fig. S15e). It failed to distinguish between intermediate and mature enterocytes, goblets and Paneth cells. It even misclassified stem, TA, k cells, enteroendocrine, and tuft cells into one cluster. The six rare clusters identified by GiniClust3 mixed with mature enterocytes and goblets cells in the UMAP embedding, suggesting they are not true rare (Additional file 1: Fig. S15e). GapClust obtained five rare clusters, two of which mapped to tuft and Paneth cells (Rare_2 and Rare_3 in the Additional file 1: Fig. S15f). The biggest rare cluster with 97 cells is goblet (Rare_5), which was not that rare. FiRE quantified 25 cells as being rare (Additional file 1: Fig. S15g). It only found 1 out of 5 tuft cells and 15 out of 28 enteroendocrine to be rare. In summary, aKNNO is far more superior than KNN, KNN_high, GiniClust3, GapClust, and FiRE, to reveal all the known abundant and rare cell types in the intestinal epithelium (Additional file 1: Fig. S15h). aKNNO is very powerful in rare cells identification, which has the ability to detect the rare Paneth cell type with only three cells.

**Fig. S15.** Application to single-cell RNAseq data from mouse intestine**.** The UMAP plot labeled by the manual annotation from aKNNO (a), KNN (b), KNN_high (c). (d) Dotplot of marker genes in clusters detected by aKNNO. (e) The UMAP plot labeled by the GiniClust3 result. (f) The UMAP plot labeled by the GapClust result. (g) The UMAP plot labeled by the FiRE result. (h) A summary of clusters identified by aKNNO, KNN, KNN_high, GiniClust3, GapClust, and FiRE.

**Section S2: Application to single-cell RNA-seq from mouse habenula**

We analyzed single-cell RNA-seq derived from mouse habenula consisting of 1,149 cells, an epithalamic brain structure critical for processing and adapting to negative action outcomes [2]. aKNNO identified 17 clusters, while KNN and KNN_high found 11 and 13 groups, respectively (Additional file 1: Figs. S16a, S16b and S16c). aKNNO found more true rare cell types than KNN and KNN_high, including clusters 11 (n=23), 12 (n=17), 13 (n=15), 14 (n=10), 15 (n=2), and 16 (n=2). They were true cell types except the cluster 16, which could be validated by cell-type specific expression of known markers. Cluster 11 with high *Gap43* and *Slc17a6*, which are only expressed in LHb neurons [2]. Cluster 12 is myoepithelium cells with high expression of *Acta2* and *Myh11*. Cluster 13 is macrophages with high *Mrc1*. Cluster 14 is fibroblasts with specific expression of *Col1a1* and *Col3a1*. Cluster 15 with only two cells, it has the high expression of *Olig1* like other oligodendrocytes (clusters 4, 5, and 8), but with specific expression of *Top2a* and *Cdc20*, suggesting it is proliferating oligodendrocyte (Additional file 1: Fig. S16d). Cluster 16 shows much lower number of UMIs and genes compared to other clusters (Additional file 1: Fig. S16e), suggesting it is a cluster with empty droplets. KNN had similar clustering with aKNNO on abundant cells but missed all the six rare clusters (Additional file 1: Figs. S16a and S16b) (clusters 11-16 in the aKNNO). Although KNN_high identified much more clusters than KNN, it also failed to detect all the six rare clusters (Additional file 1: Fig. S16c).

GiniClust3 only identified five clusters in total, which misclassified distinct cell clusters into one group (Additional file 1: Fig. S16f). All the five clusters had greater than 100 cells, meaning no rare clusters were identified by GiniClust3. GapClust detected five rare clusters (Additional file 1: Fig. S16g). Three clusters were not that rare, among of which one having 123 cells from astrocytes (cluster 3 in the aKNNO), one with 126 cells from oligodendrocytes (cluster 4 in the aKNNO), and one with 33 clusters from microglia (cluster 9 in the aKNNO). Although GapClust identified fibroblasts (cluster 14 in the aKNNO) and doublets (cluster 16 in the aKNNO), it misclassified these two clusters into one (Rare_2 in the Additional file 1: Fig. S16g). FiRE found all cells to be common (Additional file 1: Fig. S16h). In summary, aKNNO is far more superior than KNN, KNN_high, GiniClust3, GapClust and FiRE in rare cells identification (Additional file 1: Fig. S16i).

**Fig. S16.** Application to single-cell RNAseq data from mouse habenula**.** The UMAP plot labeled by the manual annotation from aKNNO (a), KNN (b), KNN_high (c). (d) Dotplot of genes marking the rare clusters detected by aKNNO. (e) The number of UMI and genes in the cluster 16 compared to other clusters. (f)The UMAP plot labeled by the GiniClust3 result. (g) The UMAP plot labeled by the GapClust result. (h) The UMAP plot labeled by the FiRE result. (i) A summary of rare clusters identified by aKNNO, KNN, KNN_high, GiniClust3, GapClust, and FiRE.

**Section S3: Application to single-cell RNA-seq from intestinal organoids**

We applied aKNNO to one single-cell RNA-seq dataset from intestinal organoids, which were isolated and grown from mice intestinal crypts [3]. aKNNO and KNN_high both identified seven clusters from the 320 cells, while KNN only found four clusters (Additional file 1: Figs. S17a, S17b and S17c). The major difference between them is that aKNNO recognized two more rare clusters, cluster 5 (n=10) and cluster 6 (n=5). Cluster 5 had specific expression of *Neurog3* and *Chgb* (Additional file 1: Fig. S17d). This cluster has been reported to be enteroendocrine progenitor cells, where Neurog3 directs the program of enteroendocrine development [4, 5]. Cluster 6 specifically expressed *Defa3* and *Defa5* (Additional file 1: Fig. S17d), which are known Paneth markers. In comparison, KNN failed to detect both two rare clusters, and KNN_high misclassified the two rare clusters into one group.

GiniClust3 identified four clusters (Additional file 1: Fig. S17f) and failed to detect rare enteroendocrine progenitor cells and Paneth cells. GapClust found two rare clusters, one with five cells and the other with two cells (Additional file 1: Fig. S17g). The one with five cells mapped to Paneth cells (Rare_2 in the Additional file 1: Fig. S17g). FiRE estimated nine cells to be rare (Additional file 1: Fig. S17h), but including only one of the five Paneth cells. The other eight cells mixed with abundant cells in the UMAP embedding, suggesting they were false. Among the two rare clusters identified by aKNNO (progenitor enteroendocrine and Paneth), only GapClust found Paneth cells, while other methods failed to detect both (Additional file 1: Fig. S17e).

**Fig. S17.** Application to single-cell RNAseq data from intestinal organoids**.** The UMAP plot labeled by the manual annotation from aKNNO (a), KNN (b), KNN_high (c). (d) Dotplot of genes marking the two rare clusters. (e) A summary of two rare clusters identified by aKNNO, KNN, KNN_high, GiniClust3, GapClust, and FiRE. (f) The UMAP plot labeled by the GiniClust3 result. (g) The UMAP plot labeled by the GapClust result. (h) The UMAP plot labeled by the FiRE result.

**Section S4: Application to spatial transcriptomics from mouse coronal posterior brain**

We analyzed a 10x Visium dataset generated from mouse coronal posterior brain including 2,797 spots (Additional file 1: Fig. S18a). Data were normalized and the top 3,000 highly variable genes were selected using SCTtransform [6]. aKNNO, KNN and KNN_high detected 26, 16, and 27 clusters, respectively (Additional file 1: Figs. S18b, S18c, and S18d). aKNNO and KNN_high both mapped dorsal hippocampus structures correctly, which involved four clusters corresponding to DG, CA3, CA1, and CA2_CA1. Their identities were supported by high expression of known markers (Additional file 1: Fig. S18e). KNN, however, only identified CA1 correctly but misclassified other regions into one cluster (Additional file 1: Figs. S18f, S18g, and S18h).

The four rare clusters (clusters 22-25 in the Fig. S18i, n <30) all lined up with specific structures. Cluster 25 had high expression of *Tmem212*, *Ccdc153* and *Odf3b*, which has been reported as ependymal cells [7]. GiniClust3 only identified nine clusters in total, which lost fine anatomical structures of the mouse brain (Additional file 1: Fig. S18j). The four rare clusters (n<30) were scattered in the spatial region. GapClust found only two rare clusters (Rare_2, n=15; Rare_1, n=6) (Additional file 1: Fig. S18k). FiRE quantified 113 rare spots, among of which only eight spots mapped to the rare cluster 25 in the aKNNO and aligned to a specific anatomical pattern (Additional file 1: Fig. S18l). FiRE also quantified smooth muscle cells as being rare, which were not that rare compared to other clusters and also detected by aKNNO (cluster 19) (Additional file 1: Fig. S18m).

We compared aKNNO with the four integrative approaches, stLearn [8], SpaGCN [9], GraphST [10], BayesSpace [11] and DR-SC [12]. We set the number of their clusters to 26, matching the cluster number of aKNNO (Additional file 1: Figs. S19a-S19f). Although they all resolved anatomical structure of the mouse brain well, aKNNO outperformed the four integrative approaches in delineating fine-grained tissue structures. For example, aKNNO successfully identified four clusters (clusters 16, 22,23,24 in the Additional file 1: Fig. S19g), which stereotyped dorsal hippocampus structures DG, CA3, CA1, and CA2_CA1. Both BayesSpace and DR-SC detected four clusters, however, it showed an unclear structure of CA3 (cluster 2 in the Additional file 1: Figs. S19h and S19l). GraphST and stLearn only found two clusters, and SpaGCN recognized three clusters that were imprecise and merged into their surrounding regions (Additional file 1: Figs. S19i-S19k).

**Fig. S18.** Application to 10x Visium spatial transcriptomics data from mouse coronal posterior brain. (a) H&E image. Spatial plot annotated by aKNNO (b), KNN (c) and KNN_high (d). (e) Dotplot of marker genes in the dorsal hippocampus structure. Spatial plot focusing on dorsal hippocampus structures annotated by aKNNO (f), KNN (g), and KNN_high (h). Spatial plot of clusters detected by GiniClust3 (i), GapClust (j), and FiRE (k). (l) A summary of rare clusters identified by aKNNO, KNN, KNN_high, GiniClust3, GapClust, and FiRE.

**Fig. S19.** Comparison between aKNNO, BayesSpace, GraphST, SpaGCN, and stLearn on the 10x Visium spatial transcriptomics data from mouse coronal posterior brain. Clustering of aKNNO (a), BayesSpace (b), GraphST (c), SpaGCN(d), stLearn(e) and DR-SC(f). Detailed view of clustering in the dorsal hippocampus structure in aKNNO (g), BayesSpace (h), GraphST (i), SpaGCN(j), stLearn(k), and DR-SC(l).

**Section S5: Application to spatial transcriptomics from mouse main olfactory bulb**

We analyzed a 10x Visium dataset generated from mouse main olfactory bulb with 1,185 spots (Additional file 1: Fig. S20a). Data were normalized and the top 3,000 highly variable genes were selected using SCTtransform [6]. aKNNO, KNN and KNN_high detected 11, 8, and 13 clusters, respectively (Additional file 1: Fig. S20b, S20c, and S20d). They all reconstructed seven-layer MOB structures well, including subependymal zone, granular cell layer, internal plexiform layer, mitral cell layer, external plexiform layer, glomerular layer, and outer nerve layer. In additional, aKNNO identified three additional clusters, one with specific expression of Ptgds (cluster 6, n=90) (Additional file 1: Fig. S20e and S20f), another with high level of Hbb-bs, Hba-a1, Hba-a2, and Hbb-bt (cluster 8, n=52) (Additional file 1: Figs. S20e and S20f), and the third one with specific expression of Tpbg (cluster 9, n=31). Cluster 6 was olfactory sheathing cells, cluster 8 was located in the glomerular layer and mapped to glomerular capillaries12, and cluster 9 has been previously reported as 5T4 granule cells13. In comparison, KNN missed all the three clusters (Additional file 1: Fig. S20g), while KNN_high failed to detect olfactory sheathing cells (Additional file 1: Fig. S20h).

GiniClust3 identified five clusters, which lost the seven-layer MOB structures (Additional file 1: Fig. S20i). FiRE found 17 rare cells (Additional file 1: Fig. S20j), all of which scattered in the mob region and didn’t map to any anatomical cell types. GapClust didn’t run successfully on this dataset. In summary, methods except aKNNO and KNN_high failed to detect any rare clusters (Additional file 1: Fig. S20k).

We compared aKNNO with the four integrative approaches, stLearn [8], SpaGCN [9], GraphST [10], BayesSpace [11], and DR-SC [12]. We set the number of their clusters to 11, matching the cluster number of aKNNO (Additional file 1: Figs. S21a-S21f). aKNNO, BayesSpace, SpaGCN, and DR-SC defined the MOB seven-layer structure well, while GraphST and stLearn failed to identify subependymal zone.

**Fig. S20.** Application to 10x Visium spatial transcriptomics data from mouse main olfactory bulb. (a) H&E image. Spatial plot annotated by aKNNO (b), KNN (c) and KNN_high (d). € Dotplot of marker genes in three rare clusters. Spatial plot focusing on the three rare clusters annotated by aKNNO (f), KNN (g), and KNN_high (h). Spatial plot of clusters detected by GiniClust3 (i), and FiRE (j). (k) A summary of rare clusters identified by aKNNO, KNN, KNN_high, GiniClust3, and FiRE.

**Fig. S21.** Comparison between aKNNO, BayesSpace, GraphST, SpaGCN, and stLearn on the 10x Visium spatial transcriptomics data from main olfactory bulb. Clustering of aKNNO (a), BayesSpace (b), GraphST (c), SpaGCN(d), stLearn (e), and DR-SC(f).

**Section S6: Identification of rare lymphatic endothelial cells from 12 pan-cancer datasets**

Endothelial cells (ECs) have been proved to promote tumor angiogenesis [13], also evolve in immune regulation in the tumor microenvironment [14]. Based on previous pan-cancer scRNAseq data analysis, ECs in tumor were classified into 5 subtypes, including ESM1 tip cells only resided in malignant tissue, ACKR1 high venous ECs enriched in tumor, CA4 capillary ECs enriched in normal tissue, FBLN5 arterial ECs and PROX1 lymphatic ECs (LECs) [15, 16]. Specifically, LECs facilitate cancer invasion in the tumor lymphatic metastasis, in which malignant cells need to squeeze between LEC junctions and move along LECs towards distant organs [17].

We applied aKNNO to identify LECs in 12 datasets across 11 cancer types [16, 18-26]. Although LECs were rare, aKNNO reported LECs in all datasets (Additional file 1: Fig. S22a). The number of LECs ranged from 16 to 158, with the percentage even less than 0.15%. For example, in the breast cancer dataset (BRCA_GSE148673), 16 out of 10,359 cells were identified to be LEC (0.15%). In the non-small lung cancer (NSCLC_EMTAB61469), 80 out of 40,218 cells were recognized as LEC (0.2%) (Additional file 1: Fig. S22a). The detailed view of clustering for each dataset was shown in Additional file 1: Figs. S23 and S24). For example, aKNNO identified two endothelial clusters in the BRCA_GSE148673 dataset, which were cluster 23 ESM1 tip cells with 107 cells and cluster 40 LECs with 16 cells (Additional file 1: Fig. S23a). Consistently, *ESM1* and *NID2* were highly expressed in the cluster 23, while *PDPN* and *PROX1* were highly expressed in the cluster 40 (Additional file 1: Fig. S23a). We estimated the percentage of LECs in the endothelial cells across cancer types (Additional file 1: Fig. S22b). Melanoma had the highest percentage of LECs, followed by UVM and HNSC, which is consistent with one previous study on pan-cancer LECs proportions [27].

**Fig. S22.** Application to 12 single-cell RNAseq data from 11 cancer types. (a) A summary of each dataset and aKNNO results. (b) Percentage of LECs in the endothelial cells across the 12 datasets.

**Fig. S23.** UMAP plots annotated by aKNNO results and expression of *PDPN* and *PROX1* marking LECs in BRCA(a), CHOL(b), CRC(c), ESCA(d), HNSC (e) and melanoma (f).

**Fig. S24.** UMAP plots annotated by aKNNO results and expression of *PDPN* and *PROX1* marking LECs in NHL_GSE128531(a), NHL_GSE147944(b), NSCLC(c), OV(d), STAD (e) and UVM (f).

**References**

1. Haber AL, Biton M, Rogel N, Herbst RH, Shekhar K, Smillie C, Burgin G, Delorey TM, Howitt MR, Katz Y, et al: **A single-cell survey of the small intestinal epithelium.** *Nature* 2017, **551:**333-339.

2. Wallace ML, Huang KW, Hochbaum D, Hyun M, Radeljic G, Sabatini BL: **Anatomical and single-cell transcriptional profiling of the murine habenular complex.** *Elife* 2020, **9**.

3. Grun D, Lyubimova A, Kester L, Wiebrands K, Basak O, Sasaki N, Clevers H, van Oudenaarden A: **Single-cell messenger RNA sequencing reveals rare intestinal cell types.** *Nature* 2015, **525:**251-255.

4. Lopez-Diaz L, Jain RN, Keeley TM, VanDussen KL, Brunkan CS, Gumucio DL, Samuelson LC: **Intestinal Neurogenin 3 directs differentiation of a bipotential secretory progenitor to endocrine cell rather than goblet cell fate.** *Dev Biol* 2007, **309:**298-305.

5. Zhu Y, Liu Q, Zhou Z, Ikeda Y: **PDX1, Neurogenin-3, and MAFA: critical transcription regulators for beta cell development and regeneration.** *Stem Cell Res Ther* 2017, **8:**240.

6. Hafemeister C, Satija R: **Normalization and variance stabilization of single-cell RNA-seq data using regularized negative binomial regression.** *Genome Biol* 2019, **20:**296.

7. Chevreau R, Ghazale H, Ripoll C, Chalfouh C, Delarue Q, Hemonnot-Girard AL, Mamaeva D, Hirbec H, Rothhut B, Wahane S, et al: **RNA Profiling of Mouse Ependymal Cells after Spinal Cord Injury Identifies the Oncostatin Pathway as a Potential Key Regulator of Spinal Cord Stem Cell Fate.** *Cells* 2021, **10**.

8. Pham D, Tan X, Xu J, Grice LF, Lam PY, Raghubar A, Vukovic J, Ruitenberg MJ, Nguyen Q: **stLearn: integrating spatial location, tissue morphology and gene expression to find cell types, cell-cell interactions and spatial trajectories within undissociated tissues.** *biorxiv* 2020.

9. Hu J, Li X, Coleman K, Schroeder A, Ma N, Irwin DJ, Lee EB, Shinohara RT, Li M: **SpaGCN: Integrating gene expression, spatial location and histology to identify spatial domains and spatially variable genes by graph convolutional network.** *Nat Methods* 2021, **18:**1342-1351.

10. Long Y, Ang KS, Li M, Chong KLK, Sethi R, Zhong C, Xu H, Ong Z, Sachaphibulkij K, Chen A, et al: **Spatially informed clustering, integration, and deconvolution of spatial transcriptomics with GraphST.** *Nat Commun* 2023, **14:**1155.

11. Zhao E, Stone MR, Ren X, Guenthoer J, Smythe KS, Pulliam T, Williams SR, Uytingco CR, Taylor SEB, Nghiem P, et al: **Spatial transcriptomics at subspot resolution with BayesSpace.** *Nat Biotechnol* 2021, **39:**1375-1384.

12. Liu W, Liao X, Yang Y, Lin H, Yeong J, Zhou X, Shi X, Liu J: **Joint dimension reduction and clustering analysis of single-cell RNA-seq and spatial transcriptomics data.** *Nucleic Acids Res* 2022, **50:**e72.

13. Jiang X, Wang J, Deng X, Xiong F, Zhang S, Gong Z, Li X, Cao K, Deng H, He Y, et al: **The role of microenvironment in tumor angiogenesis.** *J Exp Clin Cancer Res* 2020, **39:**204.

14. Yang D, Guo P, He T, Powell CA: **Role of endothelial cells in tumor microenvironment.** *Clin Transl Med* 2021, **11:**e450.

15. Zhang J, Lu T, Lu S, Ma S, Han D, Zhang K, Xu C, Liu S, Gan L, Wu X, et al: **Single-cell analysis of multiple cancer types reveals differences in endothelial cells between tumors and normal tissues.** *Comput Struct Biotechnol J* 2023, **21:**665-676.

16. Qian J, Olbrecht S, Boeckx B, Vos H, Laoui D, Etlioglu E, Wauters E, Pomella V, Verbandt S, Busschaert P, et al: **A pan-cancer blueprint of the heterogeneous tumor microenvironment revealed by single-cell profiling.** *Cell Res* 2020, **30:**745-762.

17. He M, He Q, Cai X, Chen Z, Lao S, Deng H, Liu X, Zheng Y, Liu X, Liu J, et al: **Role of lymphatic endothelial cells in the tumor microenvironment-a narrative review of recent advances.** *Transl Lung Cancer Res* 2021, **10:**2252-2277.

18. Zhang M, Yang H, Wan L, Wang Z, Wang H, Ge C, Liu Y, Hao Y, Zhang D, Shi G, et al: **Single-cell transcriptomic architecture and intercellular crosstalk of human intrahepatic cholangiocarcinoma.** *J Hepatol* 2020, **73:**1118-1130.

19. Tirosh I, Izar B, Prakadan SM, Wadsworth MH, 2nd, Treacy D, Trombetta JJ, Rotem A, Rodman C, Lian C, Murphy G, et al: **Dissecting the multicellular ecosystem of metastatic melanoma by single-cell RNA-seq.** *Science* 2016, **352:**189-196.

20. Puram SV, Tirosh I, Parikh AS, Patel AP, Yizhak K, Gillespie S, Rodman C, Luo CL, Mroz EA, Emerick KS, et al: **Single-Cell Transcriptomic Analysis of Primary and Metastatic Tumor Ecosystems in Head and Neck Cancer.** *Cell* 2017, **171:**1611-1624 e1624.

21. Pandiani C, Strub T, Nottet N, Cheli Y, Gambi G, Bille K, Husser C, Dalmasso M, Beranger G, Lassalle S, et al: **Single-cell RNA sequencing reveals intratumoral heterogeneity in primary uveal melanomas and identifies HES6 as a driver of the metastatic disease.** *Cell Death Differ* 2021, **28:**1990-2000.

22. Jeong HY, Ham IH, Lee SH, Ryu D, Son SY, Han SU, Kim TM, Hur H: **Spatially Distinct Reprogramming of the Tumor Microenvironment Based On Tumor Invasion in Diffuse-Type Gastric Cancers.** *Clin Cancer Res* 2021, **27:**6529-6542.

23. Lambrechts D, Wauters E, Boeckx B, Aibar S, Nittner D, Burton O, Bassez A, Decaluwe H, Pircher A, Van den Eynde K, et al: **Phenotype molding of stromal cells in the lung tumor microenvironment.** *Nat Med* 2018, **24:**1277-1289.

24. Gaydosik AM, Tabib T, Geskin LJ, Bayan CA, Conway JF, Lafyatis R, Fuschiotti P: **Single-Cell Lymphocyte Heterogeneity in Advanced Cutaneous T-cell Lymphoma Skin Tumors.** *Clin Cancer Res* 2019, **25:**4443-4454.

25. Gaydosik AM, Queen DS, Trager MH, Akilov OE, Geskin LJ, Fuschiotti P: **Genome-wide transcriptome analysis of the STAT6-regulated genes in advanced-stage cutaneous T-cell lymphoma.** *Blood* 2020, **136:**1748-1759.

26. Gao R, Bai S, Henderson YC, Lin Y, Schalck A, Yan Y, Kumar T, Hu M, Sei E, Davis A, et al: **Delineating copy number and clonal substructure in human tumors from single-cell transcriptomes.** *Nat Biotechnol* 2021, **39:**599-608.

27. Wang W, Wang L, She J, Zhu J: **Examining heterogeneity of stromal cells in tumor microenvironment based on pan-cancer single-cell RNA sequencing data.** *Cancer Biol Med* 2021, **19:**30-42.
