# Supplementary material for: aKNNO: single-cell and spatial transcriptomics clustering with an optimized adaptive k-nearest neighbor graph
Source: Genome Biol. 2024 Aug 1;25:203. doi: 10.1186/s13059-024-03339-y (PMC11293182; doi:10.1186/s13059-024-03339-y)
Supplement: Supplementary file 2 — Additional file 2. Review history. [file 13059_2024_3339_MOESM2_ESM.docx]

Review History

**First round of review**

**Reviewer 1**

**Were you able to assess all statistics in the manuscript, including the appropriateness of statistical tests used?**

No

**Were you able to directly test the methods?**

No

**Comments to author:**

aKNNO was developed to identify rare cell population based on adaptive kNN. aKNNO chooses k adaptively for each cell based on its local distance distribution. Benchmarking was performed on both simulated and scRNAseq data as well as a Visium dataset.

In interpreting the results, the authors showed some examples where aKNNO performed better than the other approaches. However, it lacks quantitative measure to assess the performance. This can be performed using the known annotation or on the simulated dataset if the known annotation is not perfect. The qualitative assessment has to be performed against other approaches such as GiniClust3, GapClust and FiRE.

There are some concerns about over-clustering. The author picked some examples where small clusters that aKNNO follows well with the known annotation. At the same time, aKNNO may have detected the statistical noise. This has to be assessed further for proper benchmarking.

**Reviewer 2**

**Were you able to assess all statistics in the manuscript, including the appropriateness of statistical tests used?**

No

**Were you able to directly test the methods?**

Yes

**Comments to author:**

This paper proposed an adaptive k-nearest neighbor graph to identify abundant and rare cell types simultaneously, and this method, called aKNNO, is validated to be more accurate than those typical and specifically tailored methods in identifying both abundant and rare cell types. . While aKNNO is simple but efficient in identifying rare cell types, there are certain concerns that need to be addressed.

Major:
Add the benchmarking methods specifically designed or tailored to identify rare cells in the simulation studies.
Sensitivity analysis for the paramter K_max in the proposed aKNN method.
Line 124, authors claimed that aKNNO is less sensitive to the clustering resolution. To prove it, the sensitivity analysis for the clustering resolution is required for aKNNO.
Line 399, why the filtering rule is different for mouse coronal and sagittal posterior brain, and main olfactory bulb? Please explain it and show rationale in more details.
Additionally, computational efficiency should be compared with other methods.
Some newly developed clustering methods, such as DR-SC, should be also compared as benchmark.

Minor:
Lines 425, 428-431, the format is problematic.

**Authors Response**

**Point-by-point responses to the reviewers’ comments:**

**Reviewer #1:**

aKNNO was developed to identify rare cell population based on adaptive kNN. aKNNO chooses k adaptively for each cell based on its local distance distribution. Benchmarking was performed on both simulated and scRNAseq data as well as a Visium dataset.

In interpreting the results, the authors showed some examples where aKNNO performed better than the other approaches. However, it lacks quantitative measure to assess the performance. This can be performed using the known annotation or on the simulated dataset if the known annotation is not perfect. The qualitative assessment has to be performed against other approaches such as GiniClust3, GapClust and FiRE.

***Response****: We thank the reviewer for the valuable suggestion. We have performed a qualitative assessment of aKNNO against GiniClust3, GapClust, and FiRE on the simulated datasets, revealing that aKNNO achieved the highest performance in identifying rare cells (Fig. 2 and Pages 6-7, lines 133-143).*

There are some concerns about over-clustering. The author picked some examples where small clusters that aKNNO follows well with the known annotation. At the same time, aKNNO may have detected the statistical noise. This has to be assessed further for proper benchmarking.

***Response****: We thank the reviewer for the insightful comment. We agree with the reviewer that it is important to demonstrate aKNNO's ability to identify biologically meaningful small clusters rather than statistical noise. To evaluate this, we utilized two metrics: the adjusted Rand index (AR!) and phiclust score(Mircea et al. 2022). AR!, when compared against true annotations, provides a gauge of clustering accuracy, whereas phiclust score, derived from random matrix theory, serves as a measure for identifying non-random substructure within cell clusters.*

*!n each simulation scenario, aKNNO achieved an average AR! score approaching 1 (AR!>0.995) at the resolution where the rare cluster was identified (Supplementary Fig. S1). This nearly perfect alignment with true annotations underscores aKNNO's proficiency in distinguishing biologically meaningful clusters from statistical noise. !n contrast, KNN obtained lower average AR! scores at the resolution where the rare cluster was identified (Supplementary Fig. S1). An illustrative example is provided in Supplementary Fig. S3. aKNNO identified rare NK cells without overclustering the two primary cell types (B and Naïve CD4 T cells). Conversely, KNN failed to detect rare cells at low resolution. At high resolution, although KNN recognized rare cells, it overclustered both the main Naïve CD4 T and B cells, indicating the detection of rare cells at the expense of overclustering (KNN_high in Supplementary Fig. S3). !n summary, aKNNO identifies rare cells without overclustering, whereas KNN either fails or detects rare cells at the cost of overclustering.*

*Furthermore, both simulation and real studies revealed that subcluster structures identified by aKNNO were supported by high phiclust scores, demonstrating aKNNO's ability to detect non-random substructures rather than statistical noise (Supplementary Figs. S4 and S6). For example, in the two simulated datasets from the two settings, KNN only recognized two clusters (0 and 1). Cluster 0 exhibited a high Phiclust score (∅clust=0.77 and ∅clust=0.85 in the two settings, respectively), indicating the presence of non-random substructures and the necessity for further subclustering. !n contrast, Cluster 1 (B cells) displayed a ∅clust=0, implying the absence of non-random substructure. As anticipated, aKNNO successfully subdivided cluster 0 into two subclusters without overclustering cluster 1 (Supplementary Fig. S4). We have incorporated these findings into the manuscript (Page 6, lines 126-132 and Page 8, lines 177-179) and discussed this in the Discussion section (Page 16, lines 354-356).*

**Reviewer #2:**

This paper proposed an adaptive k-nearest neighbor graph to identify abundant and rare cell types simultaneously, and this method, called aKNNO, is validated to be more accurate than those typical and specifically tailored methods in identifying both abundant and rare cell types. . While aKNNO is simple but efficient in identifying rare cell types, there are certain concerns that need to be addressed.

Major:

Add the benchmarking methods specifically designed or tailored to identify rare cells in the simulation studies.

***Response****: We thank the reviewer for the valuable suggestion. In the simulation studies, we benchmarked aKNNO against methods explicitly designed or tailored to identify rare cells, such as GiniClust3, GapClust, and FiRE. The results demonstrated that aKNNO consistently achieved superior performance in identifying rare cells compared to other approaches (Fig. 2 and Pages 6-7, lines 133-143).*

Sensitivity analysis for the paramter K_max in the proposed aKNN method.

***Response****: We thank the reviewer for this insightful comment. We have conducted a sensitivity analysis for the parameter* K*max, demonstrating that the performance of aKNNO remains consistent across varying values of* K*max (ranging from 10 to 50) (Supplementary Fig. S13). The sensitivity effect of* K*max on the aKNNO has been discussed in the Discussion section (Pages 16-17, lines 369-373).*

Line 124, authors claimed that aKNNO is less sensitive to the clustering resolution. To prove it, the sensitivity analysis for the clustering resolution is required for aKNNO.

***Response****: We thank the reviewer for the valuable suggestion. We have incorporated a sensitivity analysis at various clustering resolutions, illustrating that aKNNO is less sensitive to clustering resolution than traditional KNN for identifying rare cells (Supplementary Fig. S2). We have incorporated this result in the manuscript (Page 8, lines 117-122).*

Line 399, why the filtering rule is different for mouse coronal and sagittal posterior brain, and main olfactory bulb? Please explain it and show rationale in more details.

***Response****: Thanks for pointing this out. We did not have a specific reason to choose different filtering rules; rather, we followed filtering steps used in previous studies on the same dataset (Hu et al. 2021; Sun, Zhu, and Zhou 2020). In the revised manuscript, we explained in more detail (Page 19, lines 419-421 and Supplementary Information: Page 22, lines 200-201 and Page 25, lines 246-247).*

Additionally, computational efficiency should be compared with other methods.

***Response****: We have compared the computational efficiency of aKNNO with other methods, demonstrating that aKNNO is comparable to traditional KNN and significantly more efficient than other methods in terms of run time and memory usage (Supplementary Fig. S14). This comparison has been included in the manuscript (Page 17, lines 382-384).*

Some newly developed clustering methods, such as DR-SC, should be also compared as benchmark.

***Response****: We thank the reviewer for the valuable suggestion. Initially, we tried DR-SC in the simulation studies, but it encountered errors for half of the simulation datasets (errors in inv_sympd()). Sometimes, it even terminated the R session due to a fatal error when attempting to determine the optimal number of clusters. Subsequently, we applied DR-SC to real single-cell transcriptomics datasets. Unfortunately, DR-SC did not perform well in identifying rare cells (Fig. 1 below for review purposes).*

***Fig.1***. Performance comparison on rare cells identification for aKNNO, KNN, KNN_high, GiniClust3, GapClust, FiRE, and DR-SC in five single-cell RNAseq datasets.

Minor:

Lines 425, 428-431, the format is problematic.

*Given that DR-SC is primarily designed for spatial clustering by encouraging spatial smoothness based on a latent hidden Markov random field model, we deemed it fairer to evaluate DR-SC's performance on spatial transcriptomics data. Consequently, we benchmarked DR-SC against aKNNO and other four spatial clustering methods (BayesSpace, GraphST, SpaGCN, and stLearn) on three spatial transcriptomics datasets (Fig. 6 and Supplementary Figs. S19 and S21). In summary, DR-SC generated results similar to BayesSpace, which were satisfactory but less accurate compared to those achieved by aKNNO. Detailed results have been incorporated into the manuscript (Page 15, lines 326-330) and Supplementary Information (Page 22, lines 225-227 and Page 25, lines 266-267).*

Pancreas GSE84133

| Original | aKNNO | KNN | KNN_high | GiniClust3 | GapClust | FiRE | DRSC |
| --- | --- | --- | --- | --- | --- | --- | --- |
| macrophage | 18 (n=25) | -- | -- | 16 (n=25) | Rare_4 (n=25) | Rare (n=24) | 25 (n=31) |
| -- | 19 (n=20); doublets | -- | 23 (n=18) | -- | -- | -- | -- |
| t_cell | 20 (n=20) | -- | -- | -- | -- | -- | -- |
|  | 21 (n=17); doublets | -- | -- | -- | -- | Rare (n=17) | -- |
| mast | 22 (n=15) | -- | -- | 22 (n=15) | -- | -- | -- |
| epsilon | 23 (n=13) | -- | -- | -- | -- | -- | -- |
| -- | 24 (n=10); LECs | -- | -- | -- | -- | Rare (n=10) | -- |

| Original | aKNNO | KNN | KNN_high | GiniClust3 | GapClust | FiRE | DRSC |
| --- | --- | --- | --- | --- | --- | --- | --- |
| -- | 21 (n=23); Tle4+ neuron | -- | -- | -- | -- | -- | -- |
| -- | 22 (n=20); proliferating NPCs | -- | -- | -- | -- | -- | -- |
| -- | 23 (n=18); Nxph1+ neuron | -- | -- | -- | -- | -- | -- |
| -- | 24 (n=17); Fn1 fibroblast | -- | -- | 16 (n=11) | -- | -- | -- |
| -- | 25 (n=16); Myoc+ astrocytes | 20 (n=16) | 26 (n=16) | -- | -- | -- | -- |
| -- | 26 (n=12); Otx2+ neuron | -- | 27 (n=12) | 15 (n=12) | Rare_3 (n=12) | -- | -- |
| -- | 27 (n=7); Cd3g+ T | -- | -- | -- | -- | -- | -- |
| -- | 28 (n=5); IFN+ microglia | -- | -- | -- | -- | -- | -- |

17

10

8

20

4

2

6

7

14

5

12

13

9

3

1

18

19

16

15

21

11

Mouse brain GSM3580745

19

12

20

16

6

5 1

10

14

8 11	22

4

23

3 21

17

15

13

2

7

9

18

Mouse intestine GSM4521364

| aKNNO | KNN | KNN_high | GiniClust3 | GapClust | FiRE | DRSC |
| --- | --- | --- | --- | --- | --- | --- |
| 0 (n=143); Intermediate enterocyte | -- | -- | -- | -- | -- | -- |
| 1 (n=95); TA | -- | -- | -- | -- | -- | -- |
| 2 (n=85); Goblets | 2 (n=86) | 0 (n=86) | 2 (n=88) | Rare_5 (n=97) | -- | -- |
| 3 (n=65); Stem | -- | 3 (n=67) | -- | -- | -- | -- |
| 4 (n=62); Mature enterocyte | 4 (n=64) | 4 (n=63) | -- | -- | -- | -- |
| 5 (n=48); K cells | -- | -- | -- | -- | -- | 3 (n=51) |
| 6 (n=28); Enteroendocrine | -- | -- | -- | -- | -- | 5 (n=29) |
| 7 (n=25); Intermediate enterocyte | -- | 8 (n=29) | -- | -- | -- | -- |
| 8 (n=5); Tuft | -- | -- | -- | Rare_3 (n=6) | -- | -- |
| 9 (n=3); Paneth | -- | -- | -- | Rare_2 (n=3) | -- | -- |

| aKNNO | KNN | KNN_high | GiniClust3 | GapClust | FiRE | DRSC |
| --- | --- | --- | --- | --- | --- | --- |
| 11 (n=23); LHb neuron | -- | -- | -- | -- | -- | -- |
| 12 (n=17); Myoepithelium cells | -- | -- | -- | -- | -- | -- |
| 13 (n=16); Macrophages | -- | -- | -- | -- | -- | 15 (n=18) |
| 14 (n=10); Fibroblasts | -- | -- | -- | -- | -- | -- |
| 15 (n=2); Proliferating  oligodendrocyte | -- | -- | -- | -- | -- | -- |
| 16 (n=2); Empty droplets | -- | -- | -- | -- | -- | -- |

| aKNNO | KNN | KNN_high | GiniClust3 | GapClust | FiRE | DRSC |
| --- | --- | --- | --- | --- | --- | --- |
| 5 (n=10) | -- | -- | -- | -- | -- | -- |
| 6 (n=5) | -- | -- | -- | Rare_2 (n=5) | -- | -- |

4

6

1

7

9

2

8

5

3

Mouse habenula GSM4411753

Intestinal organoids GSE62270

1

3

UMAP2

2

4

UMAP1

9

14

8

15

13

2

6 4

7

11 1	5

3

12

10

***Response****: We have corrected the format (Page 20, lines 446, 450-452).*

**Hu, J., X. Li, K. Coleman, A. Schroeder, N. Ma, D. J. Irwin, E. B. Lee, R. T. Shinohara, and M. Li. 2021. 'SpaGCN:**

**Integrating gene expression, spatial location and histology to identify spatial domains and spatially variable genes by graph convolutional network', *Nat Methods*, 18: 1342-51.**

**Mircea, M., M. Hochane, X. Fan, S. M. Chuva de Sousa Lopes, D. Garlaschelli, and S. Semrau. 2022. 'Phiclust: a clusterability measure for single-cell transcriptomics reveals phenotypic subpopulations', *Genome Biol*, 23: 18.**

**Sun, S., J. Zhu, and X. Zhou. 2020. 'Statistical analysis of spatial expression patterns for spatially resolved transcriptomic studies', *Nat Methods*, 17: 193-200.**

**Second round of review**

**Reviewer 2**

Thank the authors for answering our previous comments. I have following comments about over-clustering issue:
The authors employ the phiclust metric to demonstrate the absence of over-clustering for aKNN. It is not clear why there appears to be a significant difference in the phiclust values between simulated data (depicted in Supplementary Figure S4) and real data (depicted in Supplementary Figure S6). In Figure S6, it is noteworthy that exceptionally high phiclust values are observed for all clusters generated by KNN. This means KNN is under-cluster, should be set a higher resolution to obtain more clusters. This also raises the suspicion that phiclust may tend to indicate under-clustering for clusters in real data analysis. It is intriguing to inquire whether aKNN clusters also exhibit similar high phiclust values. If they do, this could suggest that aKNN may be under-clustering as well. In real data cases, phiclust tends to show the under-clustering, how to use phiclust metric to judge whether is over-clustering for aKNN is not solved.

**Authors Response**

**Point-by-point responses to the reviewers’ comments:**

**Reviewer #2**:

Thank the authors for answering our previous comments. I have following comments about over-clustering issue:

The authors employ the phiclust metric to demonstrate the absence of over-clustering for aKNN. It is not clear why there appears to be a significant difference in the phiclust values between simulated data (depicted in Supplementary Figure S4) and real data (depicted in Supplementary Figure S6). In Figure S6, it is noteworthy that exceptionally high phiclust values are observed for all clusters generated by KNN. This means KNN is under-cluster, should be set a higher resolution to obtain more clusters. This also raises the suspicion that phiclust may tend to indicate under-clustering for clusters in real data analysis. It is intriguing to inquire whether aKNN clusters also exhibit similar high phiclust values. If they do, this could suggest that aKNN may be under-clustering as well. In real data cases, phiclust tends to show the under-clustering, how to use phiclust metric to judge whether is over-clustering for aKNN is not solved.

***Response****: We greatly appreciate the reviewer for pointing this out. We agree that it was puzzling to observe exceptionally high phiclust values for all clusters generated by KNN. We found that this issue occurred only in the human pancreas dataset and not in others, which had phiclust scores ranging from 0 to 1, similar to those from simulated data (Fig. 2 below and S8a-c in the manuscript). Moreover, similar to our observations from simulated data, aKNNO did not overcluster KNN-based clusters with zero phiclust values but identified subclusters in those with the highest and relatively higher phiclust values (Fig. 2 below and S8a-c in the manuscript). Additionally, subclusters identified by aKNNO exhibited distinct well-known markers, demonstrating that these subclusters are not statistical noise but biologically meaningful. The exceptionally high phiclust values in the human pancreas dataset were likely driven by hidden technical and/or biological noise. Analysis details were provided below, and the results were included in the revised manuscript.*

*As the manuscript on phiclust (Mircea et al. 2022) stated, technical and biological confounding factors can lead to high phiclust values even in the absence of meaningful subclusters. Therefore, we attempted to regress out technical/biological factors, including total counts, ribosomal gene expression, and mitochondrial gene expression. Although we obtained lower phiclust values after regressing out these factors, the values were still quite high (the updated figure with new phiclust values is shown in Fig. 1 below). Even when we increased the KNN resolution to an extremely high level (resolution=4), we still obtained extremely high phiclust values for most clusters, suggesting that there is hidden unwanted variability that we failed to regress out. Given that this pancreas dataset includes single cells collected from multiple patients, it is highly likely to contain unknown non-random technical and/or biological noise. We further examined the genes driving high phiclust values but could not identify a clear pattern or source.*

aKNNO: 9 (Endo.) +24 (Lymphatic Endo.) +21(doublets)

**KNN clusters**

aKNNO: 18 (Macro.) + 20 (T) + 22(Mast)

aKNNO: 0+10

aKNNO: 3+6+19(doublets)

aKNNO: 13 (Immune-activated stella)

+ 17 (Standard-activated stella)

*Fig. 1. Phiclust scores on kNN-based clusters for the human pancreas dataset after regression out technical and biological factors including total counts, ribosomal gene expression, and mitochondrial gene expression.*

*To determine if other datasets had the same issue, we calculated phiclust values for single-cell RNAseq datasets from mouse brain, mouse intestinal epithelium, and mouse intestinal organoid. We found their phiclust values to be similar to those from simulated data (Fig. 2 below). At the same clustering resolution, in KNN clusters with phiclust values of zero (indicating the absence of non-random substructure), aKNNO did not overcluster (Fig. 2 below). Instead, aKNNO identified subclusters in KNN clusters with the highest and relatively higher phiclust values. Additionally, in the manuscript, we demonstrated that rare subclusters identified by aKNNO were characterized by well-known marker genes and could not be detected by KNN even at very high resolutions (Fig. 4 and Figs. S15 and S17).*

*In summary, the exceptionally high phiclust values from the human pancreas dataset are highly likely to be driven by hidden technical and/or biological noise. In other single-cell RNAseq datasets, we observed similar phiclust values with those from simulated data, where aKNNO did not overcluster those KNN clusters with zero phiclust values. In contrast, aKNNO identified subclusters in those KNN clusters with the highest and relatively high phiclust values. Additionally, these subclusters exhibited high expression of well-known markers (Figs. 3-4 and Figs. S15 and S17), further demonstrating that they are not statistical noise but real cell clusters.*

*To be noted, similar to KNN, aKNNO aims to construct a k-nearest neighbor graph, allowing clustering at various resolutions. If there is potential underclustering, the aKNNO graph can be clustered at higher resolution to identify subclusters. For instance, in the case of cluster 3 exhibiting a high phiclust value in the mouse intestinal epithelium (Fig. 2c below), aKNNO did not detect subclusters at the default resolution. However, clustering can be performed on the aKNNO graph at higher resolutions to evaluate the presence of meaningful subclusters. Given that phiclust values are sensitive to technical and biological noise, the presence of high phiclust values, in conjunction with distinct meaningful markers, might serve as a reliable indicator of the existence of true subclusters.*

a	**Mouse brain GSM3580745**

aKNNO: 0+28

aKNNO: 15+22

aKNNO: 16+24

aKNNO: 6+21+23+26

KNN clusters

aKNNO: 11+17+27

b

**Mouse intestinal epithelium GSM4521364**

aKNNO: 5+6

aKNNO: 2+9

aKNNO: 0+7+8	aKNNO: 1 + 3

KNN clusters

0

aKNNO: 0+5+6

aKNNO: 3+4

c	**Mouse intestinal organoid GSE62270**

KNN clusters

*Fig. 2. Phiclust scores on kNN-based clusters for the mouse brain (a), mouse intestinal epithelium (b), and mouse intestinal organoid(c). aKNNO did not overcluster those kNN-based clusters with zero phiclust scores. In contrast, it identified subclusters in those kNN-based clusters with relatively high phiclust scores.*

*In the revised manuscript, to prevent confusion, we have replaced the original Fig. S6, derived from the human pancreas dataset, with results from datasets in the mouse brain, mouse intestinal epithelium, and mouse intestinal organoid (Supplementary Fig. S8a-S8c). Additionally, we have included a discussion highlighting that aKNNO successfully identified meaningful subclusters rather than statistical noise. This is supported by three observations: 1) aKNNO avoids overclustering in kNN-based clusters with zero phiclust values; 2) it identifies subclusters with the highest and relatively high phiclust values; and 3) these identified subclusters exhibit well-known gene markers. Furthermore, we emphasize that the primary aim of aKNNO is to construct an adaptive kNN graph accurately capturing inherent cellular structure, rather than to pinpoint the optimal clustering resolution. In fact, aKNNO can be clustered at any resolutions. Given that phiclust values are sensitive to technical and biological noise, the presence of high phiclust values, in conjunction with distinct meaningful markers, might serve as a reliable indicator of the existence of true subclusters (Page 16, 352-360).*

**References**

Mircea, M., M. Hochane, X. Fan, S. M. Chuva de Sousa Lopes, D. Garlaschelli, and S. Semrau. 2022. 'Phiclust: a clusterability measure for single-cell transcriptomics reveals phenotypic subpopulations', *Genome Biol*, 23: 18.

**Third round of review**

**Reviewer 2**

The authors answered all my concerns and I have no more quesitons.
